# A systematic review and meta-analysis on heat thresholds for maternal health and birth outcomes

**DOI:** 10.1038/s41467-026-76822-8

**Published:** 2026-08-21

**Authors:** Clara S. Heil, Leonidas G. Ioannou, Günter Alce, Susanne Frennert, Chuansi Gao

**Affiliations:** 1 Division of Ergonomics and Aerosol Technology, Department of Design Sciences, Faculty of Engineering, Lund University, Lund, Sweden; 2 School of Medicine, University of Cyprus, Nicosia, Cyprus

**Keywords:** Risk factors, Epidemiology, Outcomes research, Environmental impact, Environmental health

## Abstract

Global warming poses major public health challenges, with pregnant individuals particularly vulnerable to heat. We aim to synthesise evidence on the effects of ambient heat on pregnancy and birth outcomes and identify heat thresholds associated with increased risk of these adverse outcomes. We searched Medline, Web of Science, Scopus, GreenFile and Cinahl (June 2024) for peer-reviewed studies reporting thresholds for ambient heat; heat from medical devices, saunas, pools or studies on conception, IVF, postnatal health were excluded. Participants within studies are limited to individuals capable of becoming pregnant, including women and those identifying differently. Risk of bias is assessed using the Office of Health Assessment and Translation tool. Findings are narratively synthesised according to outcome and exposure metric, and a random effects meta-regression is used to estimate heat thresholds for acute heat effects on adverse outcomes. Evidence is limited by heterogeneity and methodological variability among studies included, and through review process limitations, including no independent dual data extraction and single person performing quality assessment. Meta-analyses determine Tmax thresholds 30.55  °C, 34.11  °C and 39.07  °C for 5%, 10% and 20% risk increase (21 studies, sample=5,589,862), suggesting increasing risk of adverse pregnancy and birth outcomes with increasing heat. The narrative syntheses (103 studies, sample=41,951,950) identifies heterogeneous indicators and a lack of comprehensive heat stress indices. Funding was received through the European Union’s Horizon Framework Programme and UKRI Innovate. PROSPERO registration: CRD42024610438.

## Abstract (web summary)

Pregnant individuals can be particularly vulnerable to environmental heat exposure. Here, the authors report a systematic review and meta-analysis that identifies temperature thresholds associated with increasing risks of adverse pregnancy and birth outcomes.

## Introduction

Climate change is the most significant global health threat of this century^1^, posing particularly high risks for adverse health and birth outcomes among pregnant individuals^2^. Heat exposure during pregnancy has been identified as a risk factor for preterm birth (PTB), low birth weight (LBW), stillbirth^3^, and other adverse health consequences, including preeclampsia^4^, congenital heart defects^5^, and placental abruption^6^. Preterm birth, defined as birth at less than 37 weeks of gestation, occurs in approximately 13.4 million infants globally each year, affecting 1 of 10 births^7^, a risk factor in more than 50% of all neonatal deaths^8^. Globally, hypertensive disorders during pregnancy occur in 3-10% of pregnancies, a leading cause of adverse pregnancy outcomes and maternal mortality^9^.

Climate change continuous to disproportionately affect women’s health, worsening existing gender inequalities, by intensifying mental health challenges, increasing risks of maternal and newborn adverse health, aggravating water scarcity, and increasing caregiving burdens^10^. While previous research has consistently demonstrated an association between heat exposure and various adverse maternal health and birth outcomes^2^, there has been limited focus on identifying specific heat thresholds at which health impacts occur. Nonetheless, establishing such thresholds is crucial, as they can be integrated into early warning systems (EWS) to alert pregnant individuals about incoming heat, enhancing risk assessments and improving health messages and guidelines and health action plans^11^. Thresholds are also important for medical and legal health considerations^12^, occupational safety regulations^13^, urban planning^14^ and for health equity strategies^15^. Heat thresholds vary depending on health outcome^16^ and heat stress indicators^11^. The wide variation in health outcomes studied and heat and health risk indicators used in previous research makes it challenging to interpret existing heat thresholds. They are developed using plain air temperature, or more comprehensive heat stress indices that account for multiple environmental and individual factors, such as the Wet Bulb Globe Temperature (WBGT), or Universal Thermal Climate Index (UTCI)^17^. For maternal health, thresholds are determined primarily based on epidemiological studies of morbidity and mortality due to the limited access of relevant physiological data.

A recent systematic review by Lakhoo et al. examined the effects of heat exposure on maternal, foetal, and neonatal health, identifying risk periods and assessing the certainty of evidence to guide policy and health interventions^18^. They specifically identified knowledge gaps on heat thresholds. While Lakhoo et al.’s review explores a wide range of health outcomes, this study narrows its focus to determining heat thresholds specifically for maternal health and birth outcomes. Another systematic review conducted by Chersich et al. highlights physiological vulnerabilities and health system challenges^19^. However, their review does not specifically address heat stress indicators in relation to the magnitude of adverse pregnancy outcomes and health risks.

In this work, we aim to synthesise evidence of heat exposure during pregnancy on health impacts and to conduct a meta-analysis to determine specific heat thresholds associated with health risks for pregnant individuals. By analysing and synthesising data on heat-health thresholds, the results of this review can provide heat-health warning thresholds, important for particularly hot climate zones, where increasing global warming may lead to regular and continuous exceedance of physiological heat tolerance limits in the coming decades^2^.

## Results

**Fig. 1:**
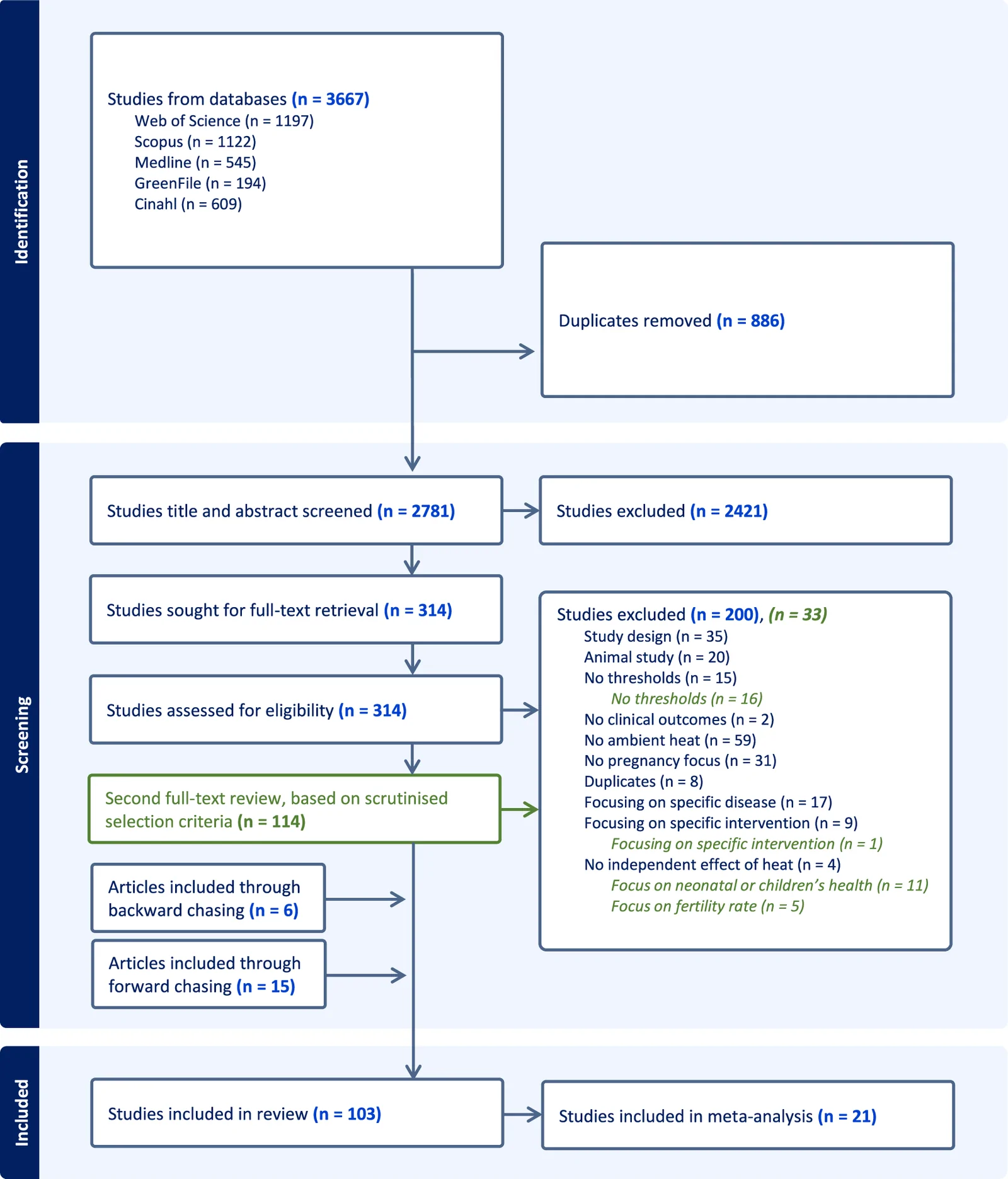
PRISMA 2020 (Preferred Reporting Items for Systematic Reviews and Meta-Analyses) flowchart, illustrating the study selection process using Covidence for literature organisation. Green, italicised categories indicate studies whose full text was reassessed after refining selection criteria.

**Fig. 2:**
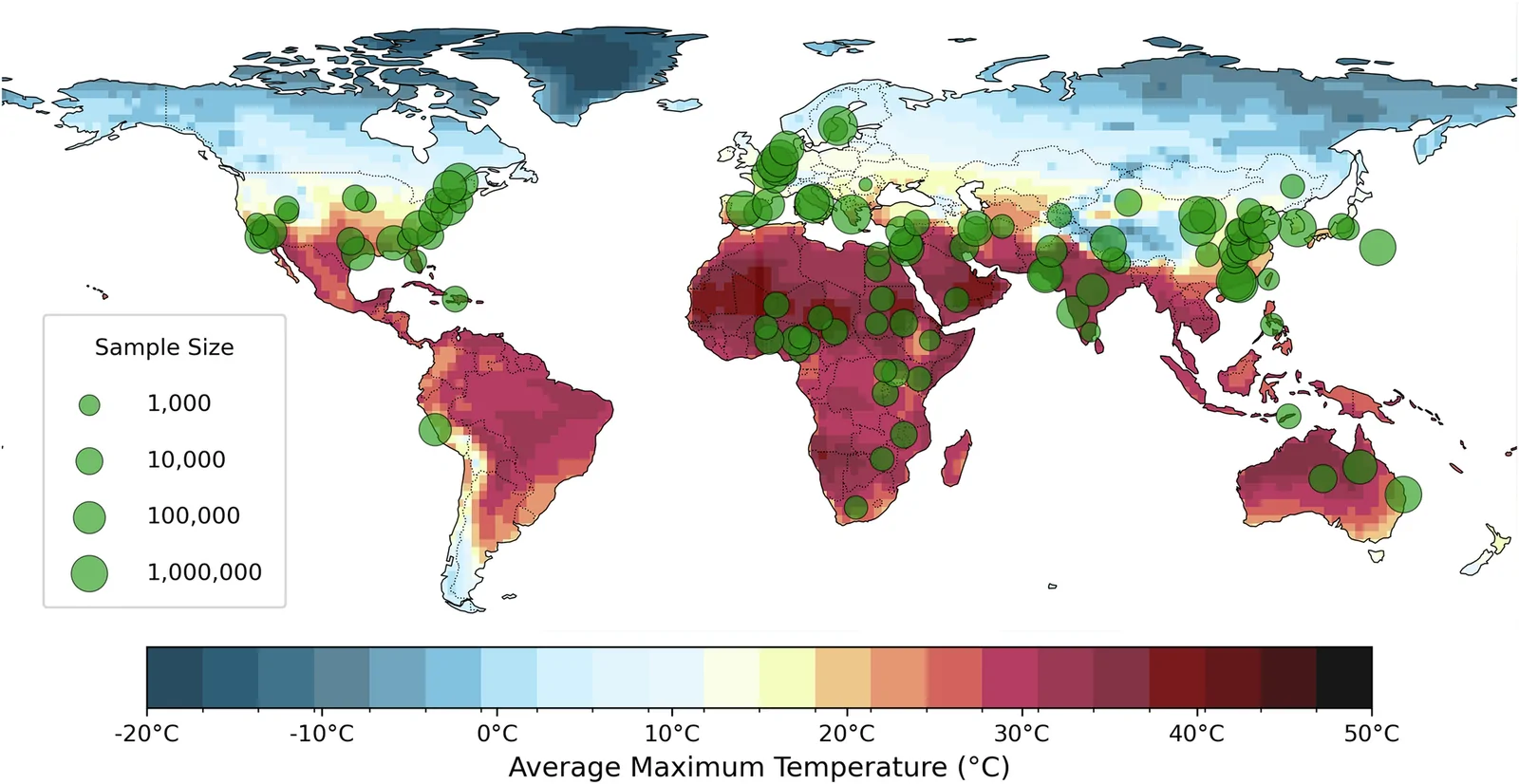
Global distribution of data extraction from the 103 eligible studies. Bubbles represent study locations where data were extracted, with some studies contributing data from multiple locations, resulting in more than 103 bubbles. The size of each bubble indicates the sample size of that specific location. If a study did not report sample sizes for individual locations but provided a total sample size across multiple locations, the per-location sample size was estimated by dividing the total sample size by the number of locations presented in the study. The background map shows the annual average of monthly maximum temperature extracted from the National Centres for Environmental Information from the National Oceanic and Atmospheric Administration (NOAA), https://www.ncei.noaa.gov/. Source data are provided as a Source Data file.

Of the 3667 studies identified for screening, we included 81 studies, 6 additional studies through backward citation chasing, and another 15 studies through forward citation chasing performed in March 2026. This resulted in a total of 103 studies for this systematic review (Fig. 1). These were published between 1999 and 2026, across six continents, with most research coming from USA (*n* = 24) and China (*n* = 24) (Fig. 2 and Table 1, Supplementary Table 1 and Supplementary Table 2 and Supplementary Table 3). Studies were conducted spanning four of five Köppen-Geiger climate classifications, including A- tropical, B- arid, C- temperate, and D- continental^20^. Only four studies cover data from low-income countries (LIC) classified according to the World Bank Atlas Method of fiscal year 2024-25^21^ (Supplementary Table 3). More than half of the studies were cohort design studies (*n* = 62), followed by case-control designs (*n* = 23), cross-sectional designs (*n* = 17), and a single human-controlled trial (Supplementary Table 7). Sample sizes ranged from 40 to 5,056,018 (Supplementary Table 6). More comprehensive information for descriptive statistics can also be found in the Supplementary Data 1.

**Table 1:** Study characteristics of 103 studies assessing heat thresholds on maternal health and birth outcomes

	Categories	Number of studies		Percent
	2025−2026		11	10.68
Year of publication	2020−2024		51	49.51
	2015−2019		28	27.18
	2010−2014		9	8.73
	2005−2009		2	1.94
	2000−2004		1	0.97
	Pre 2000		1	0.97
Geographic location	Africa		4	3.88
	Asia		42	40.78
	Europe		18	17.48
	North America		29	28.16
	Oceania		8	7.77
	South America		1	0.97
	Multiple continents		1	0.97
Health Outcome^a^ maternal health	Preeclampsia / gestational hypertension		7	6.80
	Premature rupture of membranes (PROM)		3	2.91
	Umbilical artery resistance		2	1.94
	Neural tube defects		2	1.94
	Gestational diabetes		5	4.85
	Health outcome studied by a single study		5	4.85
Health Outcome^a^	Preterm Birth		54	52.42
	Low Birth Weight		20	19.42
birth outcomes	Stillbirth		13	12.62
	Congenital anomalies / heart defects		8	7.77
	Adverse Pregnancy / Birth Outcomes (composite)		5	4.85
	Spontaneous abortions		6	5.83
	Small for gestational age (SMA)		4	3.88
	Health outcome studied by a single study		2	1.94
Heat exposure variables and environmental parameters^a^	Air temperature (Tmax, Tmean, Tmin)		80	77.67
	AT		18	15.53
	Heat Index		7	6.80
	WBGT		5	4.85
	UTCI		3	2.91

^a^The total value for some variables differs from the number of studies included when studies assessed multiple health outcomes or used multiple exposure variables. For an exhaustive list of years of publication, health outcomes, geographic locations, and heat exposure metrics used, see Supplementary Table 2, Supplementary Table 3, Supplementary Table 4 and Supplementary Table 5.

Studies assessing year of publication, geographical location, health outcomes and heat measures.

### Heterogeneity of Heat Indicators

Overall, there was considerable heterogeneity in heat indicators used across studies. Air temperature was used by 78 studies, including Tmax, Tmean, and Tmin. In total, 18 studies used apparent temperature (AT), of which 13 examined ATmax, and eight used ATmean. Five studies used the Wet-Bulb-Globe Temperature (WBGT), one reported WBGT hourly data, three reported WBGT mean, and one reported WBGTmax. Universal Thermal Climate Indicator (UTCI) was used by three studies; two reported UTCImean, and one reported both UTCImax and UTCImean (Table 1 and Supplementary Table 5).

### Summary of effects

In total, 2298 effect estimates (996 odds ratios (OR), 741 risk ratios (RR), 420 hazard ratios (HR), 120 regression coefficients, and 21 prevalence ratios (PR)) were extracted across 24 health outcomes (see Supplementary Table 4 for study count per health outcome). The direction of effect estimates was largely harmful (2155 effect estimates), with few studies (*n* = 4) reporting the opposite effect (143 effect estimates). Studies with protective effects assessed gestational hypertension (GHTN) and preeclampsia (PE)^22^, congenital anomalies^23^, PTB^24,25^, and small for gestational age (SMA)^24^, which is in line with previous research^18^. Of the 2298 effect estimates, 1183 addressed the effects of heat wave exposure. In total, 2020 effect estimates were used to conduct meta-analyses. While average temperature (Tmean) and percentiles did not yield any meaningful association with adverse maternal health and birth outcomes (OR), maximum temperature (Tmax) explained a statistically significant proportion of the variance in the risk of adverse maternal health and birth outcomes (*R*^*2*^ = 28%, *p* < 0.001). Although the overall number of ATmax studies was sufficient for a meta-regression, multiple studies did not report tangible ATmax heat thresholds or additional information required for the analysis, such as baseline heat exposure data or non-exposed controls, preventing an ATmax-based meta-regression from being conducted. Alternative heat indicators were not appropriate for the meta-analysis due to a lack of statistical power. For the Tmax meta-regression, 197 ORs from 19 studies were used after converting all effect estimates to ORs. This total reflects multiple effect estimates per study with multiple effect estimates at the same temperature level, as many studies reported results stratified by factors such as gestational period, geographic location, or several health outcomes. In the Tmax meta-regression, only lag 0 effect estimates were included due to substantial heterogeneity in lag reporting across studies. Lag effects indicate longer-term, delayed effects, which should be interpreted separately from immediate effects of heat. All lag 0 effect estimates that met the inclusion criteria described in the Methods were included in the meta-analysis. The Tmax meta-regression yielded the equation below, which defines the relationship between Tmax and the OR of experiencing adverse pregnancy and birth outcomes at any given maximum temperature.

1 $${{{\rm{Odds}}}}\,{{{\rm{Ratio}}}}\,{{{\rm{for}}}}\,{{{\rm{adverse}}}}\,{{{\rm{pregnancy}}}}\,{{{\rm{outcomes}}}}=\\ \,{e}^{0.191788-0.020604\times {{{\rm{T}}}}\max+(0.000521\times {{{{\rm{T}}}}\max }^{2})}$$

**Fig. 3:**
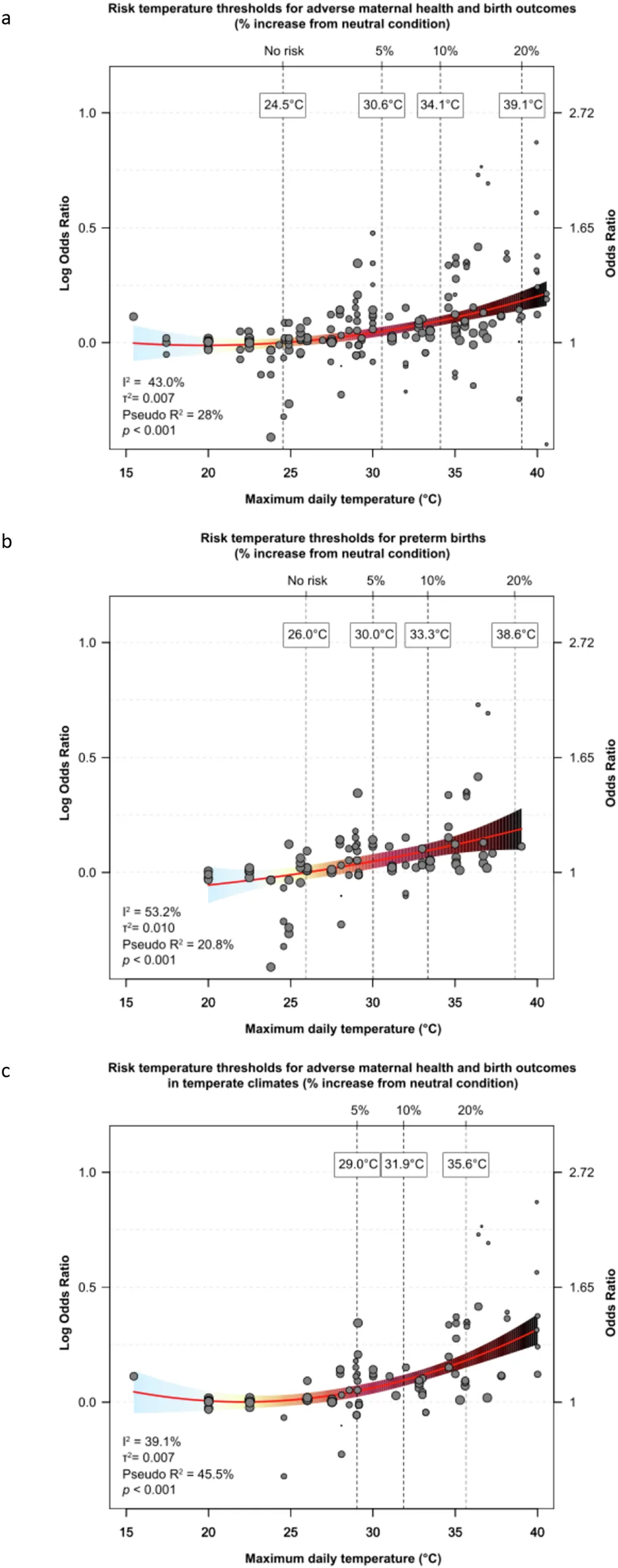
Random effects non-linear (quadratic) meta-regression analysis depicting the relationship between maximum daily temperature (Tmax) and (**a**) adverse maternal health and birth outcomes included in the 21 studies with available relevant data, (**b**) PTB included in 11 studies with available relevant data, and (**c**) adverse maternal health and birth outcomes in a temperature climate zone specifically included in 13 studies with available relevant data. Statistical tests were two-sided, and p-values for Fig. 3a-c were statistically significant (*p* < 0.001). Y-axis represents the log odds ratio (log OR), while the right Y-axis provides its corresponding odds ratio (OR). Data are presented as a black solid line, which corresponds to the predicted effect estimates from the fitted quadratic meta-regression model (random-effects). The shaded area represents the 95% confidence interval around the regression line. Each circle represents an individual study’s effect size (log OR/OR) plotted against the corresponding Tmax, with bubble size proportional to the statistical weight of each data point. Vertical dashed blue lines indicate temperature thresholds corresponding to 5%, 10%, and 20% increased risk. I2 refers to the between-study heterogeneity, τ2 represents the between-study variance, and Pseudo R2 depicts the proportion of between-study heterogeneity explained by the model. Source data are provided as a Source Data file. Supplementary Data 1 includes estimated risks for any kind of adverse pregnancy and birth outcomes included in the 103 studies from 25 to 40 °C Tmax.

At a Tmax of 30.55  °C, we determined a 5% risk increase of experiencing adverse pregnancy and birth outcomes. At 34.11  °C we identified a 10% risk increase, and at 39.07  °C, a 20% risk increase, with 24.53  °C marking no risk increase (Fig. 3a). We found a small residual, between-study heterogeneity in our meta-regression (*τ*²) of 0.007, indicating small differences in magnitude of effect estimates across studies. However, the relative proportion of remaining variability due to true between-study differences was moderate (*I*² = 43%), likely reflecting variations in population characteristics and settings, including differences in age, living conditions, and access to cooling, amongst others. We were unable to stratify the meta-analysis by gestational period due to substantial variability in definitions and assessment of gestational timing. Therefore, the results include effect estimates aggregated across all pregnancy periods. Two studies by Ren et al., included in the meta-analysis, were based on the same underlying cohort and therefore reported the same sample size, but used different methodological approaches, with the first paper assessing optimal heat thresholds for early warning^26^, while the second examined regional, socioeconomic moderators for preterm birth^27^. Health outcome specific meta-regressions were also conducted, with PTB being the only health outcome with sufficient statistical power for a meaningful result. PTB specific thresholds at 30.05  °C marked a 5% risk increase, 33.34  °C, a 10% risk increase, and 38.64  °C, a 20% risk increase. Only minor differences between PTB specific heat thresholds, compared to thresholds identified for overall adverse maternal health and birth outcome, could be determined (Fig. 3b). Furthermore, with the clear over-representation of studies from Asia, Europe, and North America in this review, with generally cooler climates, we could conduct a temperate climate zone-specific meta-regression. The climate zones were classified according to Köppen-Geigers simplified climate zone classifications^20^, representing slightly lower thresholds for adverse pregnancy and birth outcomes, with 29.03 °C marking a 5% risk increase 31.86  °C, 10% risk increase, and 35.64  °C, 20% risk increase (Fig. 3c). Heterogeneity statistics for both subgroup analyses showed small heterogeneity statistics (see Figs. 3b, 3c). No other disaggregation was possible. To ensure that the findings were not driven by a single study, we conducted leave-one-study-out sensitivity analyses by refitting the overall meta-regression after excluding each study in turn. The temperature slope remained positive and statistically significant in all runs, with thresholds varying minimally at lower threshold levels (± 2 °C) and somewhat more for higher thresholds, also reflected in the larger confidence intervals depicted in Fig. 3a below (Supplementary Data 4 for sensitivity analysis).

### Heat stress indices (HSI) and heat-health thresholds

In total, 18 studies used apparent temperature (AT), whereof 13 used maximum AT (ATmax), and eight used mean AT (ATmean). ATmax studies assessed PTB (*n* = 10)^25,28–36^, LBW (*n* = 1)^37^, adverse pregnancy outcomes (APO) (*n* = 1)^38^, neural tube defects (NTDs) (*n* = 1)^39^, and non-accidental deaths (NAD) (*n* = 1)^25^; see Table 2 for more information. Although the number of ATmax studies exceeded the minimum required for meta-analysis (*n* = 13, minimum > 10), the studies did not consistently meet the criteria required for meta-regression. Therefore, ATmax studies are described descriptively below. Eight of ten PTB studies reported an increased risk of PTB as heat increases, and two studies found no association^25,33^. The ATmax study assessing LBW reported a weak but significant association with increased heat^37^, and the NTD study found no statistically significant association^39^. For NADs, the highest risk increase reported by Kent et al. was found at the 90^th^ percentile for ≥1 day (2.0%, 95% CI 0.3, 3.8)^25^. Both protective and harmful birth outcomes were reported by van Zutphen, with the highest effect found for congenital cataracts (OR 1.45, 95% CI 1.10, 1.90)^38^. The reported PTB risk increases ranged between 15% and 22% (OR 1.15 95% CI 1.06, 1.24^40^), (HR 1.17 95% CI 1.04, 1.32, ≥40 °C vs 20  °C^34^), (OR 1.22 95% CI 0.94, 1.58 at lag 2, 36  °C vs. 31  °C^30^). Asta et al. found a large risk increase of PTB (RR 1.94, 95% CI 1.32, 2.85) at lag 0–3 for Venice, comparing the 90th to the 75th percentile of local ATmax^35^. Three studies reported PTB risk increases per 1  °C increase in ATmax, ranging from 2% to 7.1% (OR 1.02 95% CI 0.98, 3.03^29^), (OR 1.07 95% CI 1.04–1.11 for Barcelona^31^), (OR 1.07 95% CI 1.05–1.09 for Rome^31^), (OR 1.091 95% CI 0.86, 2,87)^32^, all at lag 0-2. Furthermore, a 5–7% increased risk of PTB was reported for every 10  °F (5.6  °C) at lag 5 and lag 6, and lag 06^36^. Finally, a weak association between PTB and ATmax was reported for longer-duration heatwaves (ORs ranging between 1.01–1.03, with most CIs for shorter heat durations crossing 0)^28^. ATmean was used in eight studies. ATmean studies assessed PTB (*n* = 6)^28,36,40–43^, stillbirth (*n* = 1)^44^, APO (*n* = 1)^38^, and LBW (*n* = 1)^43^. All of these found a significantly increased risk of adverse outcomes as heat increases, except when assessing LBW. Again, similarly to when using ATmax, van Zutphen found inconsistent results across adverse birth outcomes, with the highest effect reported for congenital cataracts (OR 1.47, 95% CI 1.11, 1.94). For PTB, a 4% risk increase (OR 1.04, 95% CI 1.01, 1.06) was reported overall^40^, and a 11.63% risk increase was found for every 5.6  °C ATmean increase (OR 1.12 95% CI 1.05, 1.20^42^) at lag 06. Mohammadi et al. reported the greatest risk increase of PTB at lag 0 (RR 1.61, 95% CI 1.41, 1.83), at the 99^th^ percentile of the AT distribution^41^. Similar to their ATmax findings, Huang et al. reported weak associations between ATmean and PTB during longer-duration heatwaves (OR 1.01-1.03), with most CIs for shorter heat durations crossing 0)^28^. A strong PTB risk increase was reported by Zou et al. during late pregnancy (OR 3.08, 95% CI 1.45, 6.53) at 29.63  °C. Finally, Avalos et al. reported a significant association between ATmean and PTB at lag 06 (11.6%, 95% CI 4.1, 19.7) per 10  °F (5.6  °C), higher than those reported for ATmax^36^. For stillbirth, a 10.4% increase in risk of stillbirth was found for every 10  °F (5.6  °C) increase in ATmean (95% CI 4.4%, 16.8%) at lag 2–6^44^. All AT studies were conducted in temperate climate zones following the updated Köppen-Geiger climate classifications^20^, except for one study carried out in a continental climate zone^33^, and two^30,41^ in an arid climate zone.

**Table 2:** Heat-health thresholds identified in previous literature using maximum and mean Apparent Temperature (AT) with the associated health outcomes and thresholds specified; narrative analysis of heat-health thresholds

Heat Indicator	Health Outcome	No of Studies	Description	Author	Threshold values, °C
ATmax	PTB	10	sig risk increase (*n* = 8), not significant (ns) (*n* = 2)	Cushing et al., 2022^34^ Schifano et al., 2013^32^ Asta et al., 2019^29^ Vicedo-Cabrera et al., 2014^30^ Schifano et al., 2016^31^ Asta et al., 2019^35^ Huang et al., 2021^28^ Avalos et al., 2017^36^ Porter et al., 1999^33^ Kent et al., 2014^25^	≥40 39.7 39.7 36.1 37–39 none none none 43 (ns) none (ns)
ATmax	LBW	1	sig risk increase	Yin, 2017^37^	39.44
ATmax	APO	1	sig risk increase in anophthalmia / microphthalmia, congenital cataract	van Zutphen et al., 2012^38^	44.06
ATmax	NTD	1	not significant	Soim et al., 2017^39^	35.0–36.3 (ns)
ATmax	NAD	1	sig risk increase	Kent et al., 2014^25^	none
ATmean	PTB	6	sig risk increase	Zou et al., 2025^43^ Basu et al., 2017^42^ Ha et al., 2024^40^ Mohammadi et al., 2019^41^ Huang et al., 2021^28^ Avalos et al., 2017^36^	29.63 25.06 18.1 none none none
ATmean	APO	1	sig risk increase in anophthalmia / microphthalmia, congenital cataract	van Zutphen et al., 2012^38^	21.61
ATmean	stillbirth	1	sig risk increase	Basu et al., 2016^44^	27.9
ATmean	LBW	1	not significant	Zou et al., 2025^43^	25.83–29.63 (ns)

In total, seven studies used the Heat Index (HI), assessing PTB (*n* = 4)^25,45–47^, stillbirth (*n* = 2)^47,48^, SGA (*n* = 2)^47,49^, PROM (*n* = 1)^50^, LBW (*n* = 1)^47^, and non-accidental deaths (NAD) (*n* = 1)^25^. Four of seven studies reported an increased risk of adverse outcomes with higher HI. One study found only a weak and non-significant overall association with stillbirth (OR 1.2, 95% CI 0.8, 1.6) at 33.1 °C^48^. Another study reported substantial variation across HI definitions, with null and negative associations for PTB and NAD^25^. A final study found that heat variability, rather than high heat itself, was associated with increased risk of LBW and SGA^49^. The following studies reported adverse heat-related effects. Another PTB study found a 1% to 3% increased risk associated with HI values between 32.2  °C and 36.7  °C (OR 1.01, 95% CI 1.00, 1.01), (OR 1.03, 95% CI 1.03, 1.03. No stronger associations for cumulative lags were found^45^. A risk increase in PTB during first-trimester heat exposure (HR 1.06, 95% CI 1.01, 1.11), unclear associations for the second trimester, and a strong association during third-trimester heat exposure (HR 1.51, 95% CI 1.34, 1.71) was also reported^47^. The same study assessed stillbirth and found great geographical variation, with non-significant associations in Sweden and Italy, but significantly increased risks in Belgium (HR 1.69, 95% CI 1.48, 1.92) and Greece (HR 1.26, 95% CI 1.17, 1.35)^47^. Another study reported a reduction in days of pregnancy associated with higher HI, with a mean reduction of 1.6 days (95% CI −3.2, 0.6) and a 5.3-day (95% CI −10.1, −0.5) reduction at 32  °C one day prior to delivery^46^. All studies using HI were conducted in temperate climate zones^20^, except for one conducted in a continental climate zone^49^.

Studies using WBGT (*n* = 5) assessed APO (*n* = 2)^51,52^, PTB (*n* = 2)^43,47^, umbilical artery resistance (*i* = 2)^51,53^, and foetal heart rate (*n* = 1)^51^, stillbirth (*n* = 1), SGA (*N* = 1)^47^, LBW (*n* = 1)^43^, and placental abruptions (*n* = 1)^54^. All five WBGT studies reported adverse heat-related effects. WBGT was associated with increased PTB risk during the third trimester (1.51 CI 95% 1.34, 1.71); notice the identical effect estimates to the HI analysis from this study^47^. The same study also reported increased risk for stillbirth (HR 1.54, 95% CI 1.44, 1.65) and SGA (HR 1.16, 96% CI 1.11, 1.20)^47^. Another study found strong associations between WBGT and PTB throughout pregnancy (OR 3.35, 95% CI 1.39, 8.06)^43^, as well as LBW (OR 2.89, 95% CI 1.17, 7.14). Placental abruption risk was highest at lag 1 (RR 1.23, 95% CI 1.11, 1.39). WBGT was also associated with a 13.4 beats/min increase in foetal heart rate per 10  °C WBGT increase, with WBGT values ranging between 19.3  °C and 27.3  °C (95% CI 9.5, 17.2). Rekha et al. further reported a 3.1-fold risk increase of APOs at 27.1 °C WBGT (95% CI 1.3, 7.3)^52^. Of these five studies, three were conducted in temperate climate zones^43,47,54^, one in arid^51^ and one in tropical^52^ climate zones. UTCI studies (*n* = 4) assessed PTB (*n* = 2)^47,55^, stillbirth (*n* = 2)^47,55^, umbilical artery resistance (*n* = 2)^51,53^, and LBW (*n* = 1), miscarriage (*n* = 1), preeclampsia (*n* = 1), gestational hypertension (*n* = 1)^55^, and SGA (*n* = 1)^47^. Three of four UTCI studies reported adverse heat-related effects, one found mixed results, depending on outcome^55^. One study found UTCI to be associated with increased risk of stillbirth at 46.4  °C, lag 0-13 (RR 2.049, 95% CI 1.012, 4.151)^55^, but not with PTB, LBW, spontaneous abortions, preeclampsia, or gestational hypertension. Another study reported a 10.7 beats/min increase in foetal heart rate for each 10  °C UTCI increase at temperatures ranging between 22.1  °C and 34.2  °C (95% CI 7.5, 13.8)^51^. Supporting this finding, another study, reported impaired placental-foetal blood flow, measured as changes in umbilical artery resistance (0.963 95% CI 0.981, 1.007)^53^. Of the four UTCI studies, two were conducted in a temperate climate zone^47,53^, and two were conducted in an arid climate zone^51,55^, according to the Köppen-Geiger climate classifications^20^.

### Heat thresholds for adverse maternal health outcomes

Preeclampsia (PE) and gestational hypertension (GHTN) were assessed using Tmax (*n* = 4)^22,56–58^, Tmean (*n* = 3)^22,56,59^, and UTCI (*n* = 1)^55^. Findings were mixed, with several studies reporting decreased or null associations, while others found increased risks during specific gestational periods. Using Tmean, Zhao et al. reported a significantly decreased PE/GHTN risk at a threshold of 28.7  °C (RR 0.59, 95% CI 0.39, 0.89). The same study found a similarly decreased risk using Tmax at 33.2 °C (RR 0.60, 95% CI 0.41, 0.88) at lag days 0-15^22^. Shankar et al. also reported a reduced PE/GHTN risk during first-trimester exposure to Tmax 30-40 °C (RR 0.95, *p* = 0.02), but an increased risk during the last trimester (RR 1.07, *p* = 0.005)^57^. Using UTCI and separating PE and GHTN, Khodadadi et al. found no association with GHTN, but reported an elevated PE risk at 46.4 °C UTCI (RR 1.248, 95% CI 0.872, 1.788) at lag 0-13^55^. Similarly, Tran et al. and Bogan et al. found no significant associations between Tmax and PE neither in early pregnancy (OR 1.02, 95% CI 1.00, 1.03)^58^ nor when comparing PE outpatients (OR 1.006, 95% CI 0.988, 1.023) to inpatients (OR 0.991, 95% CI 0.974, 1.008)^56^. In contrast, Part et al. reported increased PE risk associated with higher Tmean during early gestation (23 °C vs 18 °C), including week 3 (HR 1.76 95% CI 1.12, 2.78) and week 4 (HR 1.79 95% CI 1.19, 2.71)^59^. Most of these studies were conducted in a temperate climate zone, with one study extending to tropical^57^ and arid^55^ climate zones. Premature Rupture of Membranes (PROM) was assessed by three studies, using HI (*n* = 1)^50^, Tmean (*n* = 1)^60^, and Tmax (*n* = 1)^61^. All three studies reported adverse heat-related effects. Using HI, Jiao et al. increased PROM risk at 32.8  °C (HR 1.097, 95% CI 1.058, 1.138), 35.6  °C (HR 1.180, 95% CI 1.121, 1.242), 37.8  °C (HR 1.270, 95% CI 1.169, 1.380), and 40.0  °C (HR 1.456, 95% CI 1.150, 1.843)^22,50^. Using Tmean, Song et al. reported the strongest association at threshold 32  °C and lag 0–2 (RR 2.161, 95% CI 1.240, 3.764), while also lag 0–1 and 0-3 were significant, however weaker, and lag 0–4 to 0–7 were not significant^60^. Finally, PROM was associated with daily temperature variation, measured using Tmax, with higher PROM rates in hotter months, ranging between 34.1  °C and 36.2  °C^61^. Thresholds described for PROM were established for temperate^50^, continental^60^, and arid climate zones^20^.

### OHAT tool for risk of bias assessment

Although the OHAT Risk of Bias Assessment is composed of eleven questions, only seven of these were applicable to almost all studies, since four items of the tool were limited to animal studies or human-controlled trials (HCT). This review included no animal studies and only a single HCT study. The HCT study therefore required the assessment of three additional items on the OHAT tool. Two studies scored negatively on one or more items related to detection bias, confounding bias, and other biases. All other outcomes scored either + low risk of bias or ++ definitely low risk of bias, or NRs when there was a lack of sufficient information to make a judgement (Supplementary Data 3).

## Discussion

This systematic review has synthesised evidence on the impact of heat on adverse maternal health and birth outcomes, highlighting a clear trend between increased ambient heat and more adverse health outcomes. Our meta-analysis determined three Tmax thresholds: 30.55  °C associated with a 5% increase in overall adverse maternal health and birth outcome, 34.11  °C, with a 10% increase; and 39.07  °C, with a 20% increase. 24.53  °C marked no risk. For the same overall health outcome limited to a temperate climate zone, these shifted to lower thresholds at 29.03  °C at 5%, 31.86  °C at 10%, and 35.64  °C at 20%. We further conducted meta-regressions for specific health outcomes and found that PTB had sufficient statistical power for a subgroup analysis, determining three Tmax PTB thresholds at 30.03  °C for 5% increased risk, 33.34  °C for 10% increased risk, and 38.64  °C for 20% increased risk, showing slightly lower yet very similar thresholds compared to those identified for the overall health outcome. The meta-analysis identified Tmax as the most appropriate heat indicator to determine thresholds, given the available literature and sufficient statistical power required for the meta-regressions. Tmean, percentiles, and theoretically ATmax were the only other heat indicators with sufficient statistical power for meta-regressions ( > 10). However, we did not find a clear association between heat and adverse health outcomes using Tmean and percentiles, and an insufficient number of ATmax studies met the inclusion criteria required for meta-regression. Tmean is usually not used in forecasts as it is influenced by minimum temperature (Tmin). Therefore, Tmean might not be a sensitive parameter to monitor extreme weather events, and is not suitable as a heat threshold measure for EWSs.

Maternal health was addressed by less than 20% of all included studies; a gap that was also reported in a recent review by Lakhoo et al., highlighting the lack of focus on maternal health in heat-health research^18^. We conducted a narrative analysis on the impact of heat on maternal health for two maternal health outcomes: 1) preeclampsia (PE) and gestational hypertension (GHTN), and 2) premature rupture of membranes (PROM). Findings for PE and GHTN suggested heterogeneous and context-dependent temperature thresholds, with both increased and decreased risk reported across studies and gestational periods. Decreased PE/GHTN risks were observed at relatively high Tmean and Tmax thresholds 28.7  °C Tmean and 33.2  °C Tmax^22^, whereas increased risk was reported during late pregnancy at Tmax exposures of 30 to 40  °C^57^ and UTCI exposure levels of 46.6 °C^55^, and during early pregnancy at Tmean 23 °C^59^. Evidence for adverse effects was more consistently observed in early gestation and late pregnancy, although several studies reported null associations^56,58^. Lag structures were inconsistent, ranging from acute exposure, lag 0, to cumulative lag periods up to lag 15 days^22^, limiting comparability across studies^22^. The observed protective effect of heat on PE and GHTN may be attributed to its physiological effect of reducing blood pressure in the human body in the heat. From a human thermophysiological perspective, a decrease in blood pressure in response to heat exposure is a well-documented phenomenon due to vasodilation^62^, with women before menopause having particularly low blood pressure^63^. PROM findings more consistently suggested increased risk at higher heat exposure levels. Using HI, an increased risk of PROM was observed across thresholds ranging from 32.8 °C to 40.0 °C, reaching a 45% risk increase at 40.0  °C^50^. Using Tmean, the strongest association was observed at thresholds 32.0 °C^60^ during lag 0-2. Different, short lag periods remained significant but weaker, while longer lag periods (lag 0-4 to lag 0-7) became non-significant. One additional study linked higher PROM rates to Tmax thresholds ranging between 34.1  °C to 36.2  °C^50^. PROM rates appeared more acute, with stronger effects generally observed during short, cumulative lag periods.

In recent decades, over 160 thermal climatic stress indices have been developed, with more than 100 focusing on heat stress^64^. In line with Brimicombe et al.’s scoping review, the current analyses highlight the lack of a unified approach when assessing the effects of heat on pregnant individuals^65^. As heat variables interact and heat stress results from their interplay, it is important to assess the combined effect of thermal environment parameters. Only around 32% of studies use integrated heat stress indices (HSI), a comprehensive heat stress measure that includes the integration of multiple parameters in its heat stress estimation. Most heat indicators are designed based on the physiology of young, healthy men^66^, whose thermophysiological responses differ from those of pregnant individuals. Research suggests that the WBGT, an international standard for heat-stress screening, is the most sensitive heat-health indicator, particularly for vulnerable populations^11^. More research is, however, needed to determine the most suitable heat indicator for pregnant individuals. Our review emphasises that more consistency in the use of heat indicators and a shift towards integrated heat indicators are needed.

Effective heat warning systems for pregnant individuals should establish different warning levels based on the severity of health outcomes. This review identified three levels of heat warnings with respective thresholds at 30.55  °C, 34.11  °C, and 39.07  °C for 5%, 10%, and 20% increases in adverse pregnancy and birth outcomes. Thermophysiological heat strain, marked by body responses such as increased sweating and body temperature, dehydration, dizziness, headaches, and muscle cramps, occurs at lower and shorter heat exposures, and therefore requires earlier warnings and lower heat thresholds compared to clinical outcomes. In contrast, clinical health risks, such as adverse pregnancy outcomes, typically emerge at higher heat thresholds with longer exposure time and should therefore authorise separate high-risk alerts. The MotherHeat Alert is a mobile-based EWS currently developed within the European HIGH (Heat Indicators Global Health) Horizon project: https://www.high-horizons.eu/. It is specifically designed to warn and advise pregnant and postpartum women (and maternal health workers) of upcoming heat events. At present, the warning thresholds for this EWS are based on preliminary results from internally collected data, as there is no sufficient data from the respective countries to identify suitable thresholds. Although our review synthesises threshold information into global thresholds, these findings can inform systems such as the MotherHeat Alert by providing initial approximations of suitable heat thresholds. The identified levels of risk increase can serve as a guide for defining different levels of urgency in heat warnings, and are particularly applicable to preterm birth outcomes and adverse pregnancy and birth outcomes in a temperate climate.

Research has started assessing thermophysiological triggers for adverse clinical maternal health and birth outcomes, such as reduced placental blood flow, intrapartum maternal fever, dehydration, and uterine contractility, all of which need to be explored further to deepen our understanding and to find a suitable early warning threshold^67^. With a greater understanding of the physiological responses of pregnant individuals, early warnings could be issued based on thermophysiological effects and clinical implications. Furthermore, current early warning systems (EWS) and policies are developed primarily considering heat intensity to determine thresholds, although duration of exposure also seems to be a critical factor^68^. Efforts have been made to integrate exposure duration into risk calculations for EWS. Nevertheless, more research is needed to explore how heavily exposure duration should be weighted, compared to exposure intensity, to find the optimum warning threshold^68^. Finally, it is essential that an effective warning system strikes a balance when issuing warnings. Frequent warnings will likely be ignored, reducing their effectiveness, whereas warnings issued too late will fail to protect individuals from harm^69^.

Research largely agrees that high ambient temperatures have adverse effects on pregnancy outcomes^2^, and that pregnant individuals are considered particularly vulnerable to heat exposure^67^. Prior reviews underline the adverse impacts of rising global temperatures on maternal and neonatal health, focusing on LIC, where climate change effects are disproportionately severe^18,19^. Our review highlights a geographical imbalance in research on heat exposure during pregnancy. While we imposed no restrictions on date or location during literature selection, most studies originate from high- and upper middle-income countries, with data from LICs assessed by four studies only, covering The Gambia^51^, Burundi, Ethiopia^70^, Benin^71^, Mali, Nigeria, Zimbabwe, Madagascar, Senegal, Kenya, Lesotho, Burkina Faso, Guinea, Rwanda, Zambia, Sierra Leone and Liberia^72^ and Malawi and Uganda^70,72^. This imbalance likely leads to an underestimation of risk experienced in hotter regions. Consequently, the generalisability and equity of this review are limited. Populations in resource-limited settings often face higher baseline heat exposure and reduced adaptive capacity, including access to cooling, healthcare, and EWS, and may therefore experience greater risks than our estimates suggest. Subgroup analyses could only be conducted for temperate climate zones, further highlighting the geographic imbalance and the urgent need for more research from hotter, low- and lower-middle-income regions to improve global applicability. This lack of global research coverage, particularly in the Global South, has also been identified by Lakhoo et al. and Chersich et al. Both reviews stress the urgent need for developing and implementing solutions to protect pregnant individuals from escalating temperatures^18,19^. Effective interventions must be optimised to each setting and tailored to local contexts, co-designed with key stakeholders, including pregnant and postpartum women, healthcare workers, and policymakers^19^. However, the lack of regional diversity makes it difficult to establish heat risk thresholds that reflect local and global climatic conditions. Furthermore, the above-mentioned reviews explicitly state the need for developing EWS to mitigate heat-related pregnancy complications^19^, determining clear heat-health thresholds for suitable warning triggers^18^. More research from the Global South is needed to ensure that heat thresholds are applicable across diverse settings.

This systematic review has various limitations. Despite efforts to include all relevant studies to determine heat risk thresholds for maternal health and birth outcomes, some may still have been missed. We further acknowledge that the selection of databases searched likely biases the results towards English-language articles and may have reinforced publication biases. Furthermore, the definitions of heat and health used in this review could be different from definitions used elsewhere, since we deliberately narrowed these, which may have resulted in fewer included studies (see data analysis and exclusion criteria in methods). In addition to the predefined limitations of our heat variable, we did not assess minimum temperature (Tmin). Although Tmin is an important indicator of heat stress through nighttime cooling and recovery^73^, our review prioritised evaluating exposure to hot days, rather than hot nights or heat fluctuations. By restricting the scope to both heat and health variables, this review ensures a more precise focus on the impact of ambient heat on maternal health and birth outcomes, yet acknowledges that studies relevant to neonatal health, conception rates, and more controlled heat conditions and other heat parameters have been excluded. Although information on covariates was collected, we did not explicitly control for these in our analysis. Furthermore, risks of bias may have been introduced directly by the authors, through design flaws, data availability or selective reporting, or subjective evaluation of bias in the quality assessment. Despite negative evaluations of two studies on the OHAT assessment, we included all studies in the review. Including studies with varying levels of research rigour may nonetheless have introduced differences in study quality and consequently influenced the analysis results. Additionally, although most of the study selection processes were performed by two independent reviewers, biases may still have been introduced specifically in stages with a single reviewer, including the title and abstract screening phase, and in the risk of bias assessment, where additional reviewers provided continuous feedback and were consulted in case of uncertainties, but did not independently complete assessments.

While the review included 103 studies, the overall Tmax meta-regression was limited to 21 studies for the aggregated health outcome, 11 studies for the PTB specific meta-regression, and 13 studies for the aggregated health outcome in the temperate climate zone. In our meta-analysis, we used OR as our effect statistic. Although all RR, HR, PR, and Regression Coefficients could be converted into ORs, not all Tmax data points could be used in the meta-regression, since, due to high heterogeneity, we excluded effect estimates that were based on lag days, making our meta-regression specific to lag 0 outcomes. We acknowledge that excluding lagged estimates omits delayed temperature effects; however, to ensure methodological consistency across studies, this approach was chosen. Heat exposure data analysis excluded duration and frequency of exposure, the distinction between heat waves and other heat exposures, and heat variability measures. Future reviews should standardise and incorporate these measures to improve the accuracy and robustness of findings.

Our aim with this review was to address the threshold gap by systematically synthesising the evidence-based heat health associations and heat thresholds determined by previous studies. We demonstrated that heat thresholds for adverse maternal health and birth outcomes can be determined based on maximum temperature, with 30.55  °C, 34.11  °C, and 39.07  °C serving as thresholds for 5%, 10%, and 20% risk increase of experiencing overall adverse pregnancy outcomes. Due to the high heterogeneity amongst the studies, it was only possible to establish two disaggregated sets of thresholds, one for the overall adverse pregnancy and birth outcome in a temperate climate zone, and one for all climate zones but PTB specifically. Broad associations between heat thresholds and maternal health outcomes were identified through a narrative analysis and may be useful before a more robust meta-analysis with sufficient data can be carried out. We have further demonstrated that future research should focus on integrated HSI that accounts for multiple factors that influence an individual’s response to heat exposure. Furthermore, greater emphases should be placed on lagged effects, and duration and frequency of heat exposure, as these are key parameters in assessing the health impacts of heat. Finally, future research should adopt a more standardised reporting practise on individual and environmental factors that influence the experience of heat exposure, such as age, acclimatisation, activity level, access to cooling, socio-economic status, humidity, solar radiation, and wind speed, amongst other parameters. This way, more accurate heat thresholds can be determined, upon which context-specific and actionable public health interventions can be developed. Researchers should work closely with policymakers and health workers to develop and implement EWS for pregnant individuals, supporting the UN’s Early Warnings for All (EW4All) initiative, which, by 2027, aims to provide life-saving early warning access to all, including vulnerable populations^74^. The EWS should incorporate a warning threshold based on both intensity and duration of heat exposure, and on different predefined warning levels of both physiological strain and clinical risk, to effectively mitigate adverse health consequences.

## Methods

### Definition of heat, heat threshold, heat indices, and adverse maternal health and birth outcomes

Our heat variable was limited to ambient, outdoor heat. Literature examining heat induced through specific medical devices, interventions, saunas, or swimming pools was excluded, since these studies typically focus on instantaneous thermoregulatory responses and behavioural adaptations, making it difficult to compare to the effects of ambient heat exposure on long-term clinical adverse health outcomes. Different from localised, short-term heat exposure, ambient heat can persist for an entire day or extend across multiple days and weeks during an extreme heat event (EHE). In this review, we only assessed the acute effects of ambient, outdoor heat on adverse health outcomes in the meta-analysis and therefore focused exclusively on lag 0 (same-day exposure) effects. For the narrative review, when several significant outcomes were reported, we consistently extracted the highest effect estimates, regardless of lag structure. Whenever an effect estimate was linked to a certain lag structure, this was explicitly reported. Heat thresholds in the context of health represent critical heat levels -potentially including other environmental parameters such as solar radiation or humidity- at which mortality and morbidity rates increase significantly. For health outcomes, we considered maternal health to be strictly confined to any complications during pregnancy, not focusing on the influence of heat on conception rates and IVF patients. Birth outcomes were limited to outcomes at the time of birth, excluding any neonatal health consequences that may arise following. Furthermore, in the field of heat and health, adverse pregnancy outcomes (APO) are sometimes referred to as a composite score that typically assesses stillbirth, PTB, LBW, miscarriage, and congenital abnormalities^52^, however, the exact inclusion of adverse health outcomes may differ across studies. Furthermore, as this review focused on adverse pregnancy and birth outcomes, only studies involving pregnant individuals were included. Sex was therefore inherently considered in the study design, and sex disaggregated analyses were not applicable. Finally, as covariates were included only as secondary variables to provide contextual insights into study demographics, we categorised them as i) analysed (controlled for in main analysis), ii) reported (presented descriptively but not used as covariates), or iii) not reported. Information on gestational period was harmonised into trimesters, as studies differed in reporting this variable by gestational weeks or trimesters (Supplementary Data 2 for details).

Exposure variables have been measured using different heat indicators and conditions. Heat can be assessed using plain air temperature, measuring for instance Tmax, Tmean, and Tmin, which are widely used parameters since they are simple and straightforward to understand. In contrast, integrated heat stress indices (HSI) are more complex as they measure several factors. They include, for instance, Apparent Temperature (AT), Heat Index (HI), the WBGT (Wet Bulb Globe Temperature), and UTCI (Universal Thermal Climate Index). Both AT and HI assess how hot it feels under certain conditions. AT is a measure of relative discomfort and assesses both heat and cold perception, incorporating air temperature and humidity^64^. HI is used to assess heat risk through air temperature and humidity combined^64^. WBGT is a screening heat index^75^ that was originally designed for military settings, but is now one of the most widely used occupational heat stress index^76^. Next to air temperature and humidity, it also considers radiant temperature and air velocity, with the most updated version including activity intensity and clothing effect as additional parameters^77^. The UTCI is designed to measure thermal stress in outdoor environments aiming to promote public health^75^. Like the WBGT, it also includes air temperature, radiant temperature, humidity, and air velocity, and it considers human thermoregulation. Nevertheless, UTCI covers a wider range, assessing both hot and cold thermal climates^78^. Heat variability is typically reported as a diurnal temperature range (DTR), indicating the difference between Tmin and Tmax, or an interquartile range (IQR). It can be reported in terms of maximum day-to-day temperature, either as an average or a standard deviation of a specific time-period^79^. Finally, heatwaves influence human health and wellbeing and are therefore often assessed in heat-health research^80^. Heatwaves refer to acute periods of extreme warmth; however, there is no single, universally consistent heatwave definition^81^. Generally, heatwave definitions differ along three criteria, including the temperature metric, intensity and temperature threshold, and duration, yet two consecutive days of abnormally high heat is often referred to as the minimum requirement for a heatwave^80^.

### Search strategy and selection criteria

We conducted this systematic review and meta-analysis according to the Meta-analysis of Observational Studies in Epidemiology (MOOSE) guidelines^82^, and followed the Preferred Reporting Items for Systematic Reviews and Meta-Analyses (PRISMA) 2020 as reporting guidelines^83^, which can be found in the supplementary information file (Supplementary Note 1 for PRISMA 2020 abstract checklist, Supplementary Note 2 for PRISMA 2020 checklist). We did not apply any date or language restrictions. EndNote21 was used to export search results, and Covidence was used to organise the literature selection process, separating relevant studies from non-relevant studies. Original peer-reviewed studies that determined a heat threshold at which adverse maternal health and birth outcomes occur were included. All study designs were included except for reviews, editorials, and reflections. No grey literature, trial registries, or unpublished data were included, and we did not contact any authors for additional information. This review was registered in PROSPERO (registration ID: CRD42024610438). No review protocol was developed, and no amendments were made to the registered information. Studies were excluded if they were animal or plant studies, contained no heat threshold, did not focus on pregnancy or ambient heat, focused on conception and IVF patients, hypothermia, the combined effect of pollution and heat (no individual heat effect), hospital admissions without clear clinical health outcomes, or if they focused on neonatal or child health only.

Covidence automatically removed 848 duplicates. Title and abstract screening was performed on 2781 studies, whereof 314 studies were selected for full-text review. These were assessed by two independent authors. Finally, 114 studies met the initial selection criteria. A second round of full text review was, however, conducted after scrutinising the selection criteria, rendering the 81 studies for final inclusion (Fig. 1 and Supplementary Data 1). The search was carried out across five databases: Scopus, Web of Science Core Collection (for more precise information on journals included, see Table 1 in the appendix), Medline (PubMed), CINAHL, and GreenFile, covering purely health, nursing, and environmental journals as well as more general ones. Search terms used were: heat*, thermal, warm*, temperature, threshold, limit, indicator, max*, warning, alert, monitor, maternal, pregnan*, postpartum*, perinat*, “peri nat*“, prenat*, “pre nat*“, postnat*, “post nat*“. Boolean operators AND and OR were used to group keywords into three distinct concepts, namely 1) heat, 2) threshold, and 3) pregnancy (see appendix Table 1). These search terms were derived following a pilot keyword search using Scopus and Web of Science Core Collection. In this “title only” search, we used preliminary keywords to ensure that highly relevant papers appeared. The papers were assessed for their keywords, resulting in an iterative process of keyword revision. Together with a librarian, we explored the MeSH hierarchy in PubMed to assess subject headings and their automatic explosion, in order to identify the most suitable terms for our search. We made final adjustments to the keywords after completing both processes. The final, comprehensive search strings can be found in Appendix 1. Furthermore, the “heat” search terms were connected with the “threshold” search terms using a proxy delimitator, restricting a maximum of “n” words between any word from the respective concepts. This way, we increased the likelihood of linking the concept “threshold” to the concept “heat”, since numerous irrelevant studies appeared if this procedure was not undertaken. We selected a proxy delimiter of n = 6, following additional search rounds and consultations between authors and a librarian. This proxy delimiter was selected since n = 6 yielded many relevant results without excluding key papers identified within the first 30 results. Seven studies with restricted access to the full text were requested and received through university librarians. Furthermore, backwards citation chasing was performed by hand-searching the reference lists of included studies, identifying six additional articles. Forward citation chasing was also conducted using Google Scholar, as recommended by the librarian due to its broad source coverage. To determine eligibility during forward and backward citation chasing, the threshold concept was screened within the title and abstract, consistent with the main search strategy. The forward citation-chasing search identified 15 additional studies for inclusion, of which four had been published before the original search date (June 2024), supporting the robustness of the original search strategy. Additionally, a tertiary-level course on systematic review methodology was completed to ensure a thorough and standardised approach to the analysis.

### Data analysis

Title and abstract screening was carried out by the first author. For the full-text screening, both the first author and another co-author, conducted independent assessments. Inter-rater discrepancies in study selection for inclusion were resolved in several discussion sessions between the two reviewers and other co-authors of this review. Data extraction was piloted on five included articles, during which the extraction criteria were refined until discrepancies were resolved. The agreed-upon framework was then applied to the remaining studies by the first author. Following data extraction by the first author, the data were fully checked by a second author. Weekly discussions during data extraction were held to address uncertainties and ensure alignment. Data extraction was separated into two steps using Excel, with several iterations within these two steps. In the first step, we extracted data on study design, location, sample size, main study outcomes, including heat and health variables, exposure information, and covariates. The second step simplified the information extracted in the first step into a more numerical form useful for quantitative analysis. For data extraction specific to the meta-analysis, continuous support and feedback from the second author, responsible for the quantitative data analysis, were provided. The meta-analysis was performed on studies using Tmax, Tmean, or percentiles as the heat indicators, due to the lack of sufficient suitable data for other heat indicators. Studies that did not report on non-exposed controls, baseline heat exposure data, tangible heat thresholds, or inconsistent heat indicators between baseline and exposure conditions were excluded, since this is required to calculate the OR. Therefore, no meta-regression could be conducted using ATmax results. The review focused on summary estimates, as increases in health risks due to heat exposure were assessed across populations. For non-numeric information presented only graphically in included studies, such as confidence intervals depicted in visualisations, values were extracted using WebPlotDigitizer 5.2^84^. The narrative analysis assessed different heat indicators used by the included studies to identify thresholds. It further explored associations between heat exposure and health outcomes for which suitable or sufficient data for a quantitative analysis. For both the meta-analysis and the narrative analysis, we consistently extracted effect estimates from the most-adjusted models. We used the OHAT tool, developed by the Office of Health Assessment and Translation (OHAT) under the National Toxicology Program (NTP), a systematic framework for assessing risk of bias, particularly suitable for environmental health research^85^. The 11-item tool addresses issues related to selection, confounding, and detection biases, amongst other aspects. Ranging from ++ indicating definitely low risk of bias to -- indicating definitely high risk of bias, four answering options are available for each item. The first author conducted the quality assessment for most studies, except for two studies published in a different language, which were assessed by a coauthor fluent in that language.

### Effect size conversion

To ensure a uniform effect size across all studies, we converted all reported effect sizes that were not Odds Ratios (ORs), including Risk Ratios (RRs), Hazard Ratios (HRs), Prevalence Ratios (PRs), and Regression Coefficients, to ORs. RRs, HRs, and PRs were converted to ORs assuming a small baseline risk (7.5%). This baseline risk was derived from published prevalence estimates of PTB and LBW in high-income countries, where PTB does not exceed 8%^7^, and LBW is reported at 7.2%^86^. PTB and LBW were the most common health outcomes in our meta-analysis, accounting for 12 of 21 studies. Given that the majority of studies were conducted in high-income settings, the 7.5% baseline risk is a reasonable approximation. This approach is further supported by another health related meta-analysis that has applied the same baseline risk^87^, using the formula^88–90^:

2 $${{{\rm{OR}}}}=\frac{{{{\rm{RR}}}}\times (1-{{{{\rm{P}}}}}_{0})}{1-{{{\rm{RR}}}}\times {{{{\rm{P}}}}}_{0}}$$

where ${P}_{0}$ is the baseline risk and represents the proportion of unexposed individuals who experienced any kind of adverse pregnancy outcomes in the included 103 studies. When RRs, HRs, or PRs were not provided, but Regression Coefficients were available, they were exponentiated as follows to obtain ORs^91^:

3 $${{{\rm{OR}}}}={e}^{{{{\rm{Regression}}}}{{{\rm{Coefficient}}}}}$$

We performed a baseline-risk (*P*_0_) sensitivity analysis by re-estimating the overall meta-regression across *P*_0_ values from 0.025 to 0.15. The temperature slope remained positive and highly significant in every run with only minor changes in magnitude (Supplementary Data 5). Threshold estimates were similarly robust across all baseline assumptions: on average, 5% risk increase thresholds varied by ±0.06  °C, 10% by ±0.12  °C, 15% by ±0.17  °C, and 20% by ±0.22  °C relative to *P*_0_ = 0.075. These results indicate that the substantive conclusions are not sensitive to the assumed baseline risk.

### Calculation of standard errors

Standard Errors (SE) for the log-transformed ORs were calculated using the provided lower and upper confidence intervals where available, using the following formula^92,93^:

4 $${{{\rm{SE}}}}=\frac{\log \left({{{{\rm{OR}}}}}_{{{{\rm{upper}}}}\,\,{{{\rm{bound}}}}}\right)-\log ({{{{\rm{OR}}}}}_{{{{\rm{lower}}}}\,\,{{{\rm{bound}}}}})}{2\times 1.96}$$

For missing SE values that could not be calculated due to a lack of confidence intervals in the study, we imputed SE using the average Coefficient of Variation (CV) derived from available data for the same heat stress indicator^94,95^.

### Meta-regression analysis

Using the calculated ORs and SE values, linear and non-linear (quadratic) meta-regressions were conducted using a random-effects model (REML) to account for the between-study heterogeneity resulting from different protocols, geographies, and populations. Linear and non-linear relationships between heat indicators, with suitable data from more than 10 studies and the log odds ratios (log OR) were calculated. The models included temperature as both linear and non-linear terms. Our study presents a model that better explains the variance of adverse pregnancy and birth outcomes due to heat exposure. Tmax thresholds indicating 5%, 10%, and 20% increase were derived by solving the regression equation. All analyses were conducted using the “metafor” package in R language (Rstudio, Version 1.3.1093, PBC, Boston, Massachusetts, United States)^96^. The level of significance was set at an alpha of *p* < 0.05.

### Reporting summary

Further information on research design is available in the Nature Portfolio Reporting Summary linked to this article.

## Supplementary information

Supplementary Information

Description of Additional Supplementary Files

Supplementary Dataset 1

Supplementary Dataset 2

Supplementary Dataset 3

Supplementary Dataset 4

Supplementary Dataset 5

Reporting Summary

Appendix1

Transparent Peer Review file

## Source data

Source Data 1

## Data Availability

Source data are provided with this paper. Data extracted from previously published studies are available in Supplementary Data 1-2. The raw data underlying the Tmax meta-regressions and the corresponding analysis outputs have been deposited in Figshare repository under accession code: 10.6084/m9.figshare.32802512^97^. Additional study characteristics, risk-of-bias assessments, and sensitivity analyses are provided in the Supplementary Information and Supplementary Data files. There was no custom code developed for the study analyses. Source data are provided with this paper.
