# Supplementary material for: A systematic review and meta-analysis on heat thresholds for maternal health and birth outcomes
Source: Nat Commun. 2026 Aug 21;17:8768. doi: 10.1038/s41467-026-76822-8 (PMC13493939; doi:10.1038/s41467-026-76822-8)
Supplement: Supplementary file 1 [file 41467_2026_76822_MOESM10_ESM.pdf]

APPENDIX 1: Search keywords and search string

(Table 1) Information sources, search string and results. Search conducted May 10, 2024.

Journal	Field of Search	Proxy Delimiter	Search String	Filter	Results Yielded	Results for Screening (duplicates removed)
Scopus	Article title, Abstract, Keywords	W/6	(heat* OR thermal OR warm* OR temperature) W/6 (threshold OR limit OR indicator OR max* OR warning OR alert OR monitor) maternal OR pregnan* OR postpartum* OR perinat* OR "peri nat*" OR prenat* OR "pre nat*" OR postnat* OR "post nat*"	none	1122	1121 (1)
Medline, PubMed	All text	N/6	(heat* OR thermal OR warm* OR temperature) N/6 (threshold OR limit OR indicator OR max* OR warning OR alert OR monitor)<br><br>maternal OR pregnan* OR postpartum* OR perinat* OR "peri nat*" OR prenat* OR "pre nat*" OR postnat* OR "post nat*"	humans	545	248 (297)
Web of Science Core Collection*	Topic	NEAR/6	(heat* OR thermal OR warm* OR temperature) NEAR/6 (threshold OR limit OR indicator OR max* OR warning OR alert OR monitor)<br><br>maternal OR pregnan* OR postpartum* OR perinat* OR "peri nat*" OR prenat* OR "pre nat*" OR postnat* OR "post nat*"	none	1197	647 (550)
GreenFile	All fields	none	(heat* OR thermal OR warm* OR temperature) AND (threshold OR limit OR indicator OR max* OR warning OR alert OR monitor) maternal OR pregnan* OR postpartum* OR perinat* OR "peri nat*" OR prenat* OR "pre nat*" OR postnat* OR "post nat*"	none	194	168 (26)
Cinahl	All fields	none	(heat* OR thermal OR warm* OR temperature) AND (threshold OR limit OR indicator OR max* OR warning OR alert OR monitor) maternal OR pregnan* OR postpartum* OR perinat* OR "peri nat*" OR	none	609	597 (12)

			prenat* OR "pre nat*" OR postnat* OR "post nat*"			

*Web of Science Core Collection includes Science Citation Index Expanded (SCI-EXPANDED), Social Sciences Citation Index (SSCI), Emerging Sources Citation Index (ESCI), Conference Proceedings Citation Index Science (CPCI-S), Book Citation Index – Science (BKCI-S), Arts & Humanities Citation Index (A&HCI), Conference Proceedings Citation Index – Social Science & Humanities (CPCI-SSH), Book Citation Index – Social Sciences & Humanities (BKCI-SSH), Index Chemicus (IC), Current Chemical Reactions (CCR-EXPANDED)
