# Supplementary material for: A systematic review and meta-analysis on heat thresholds for maternal health and birth outcomes
Source: Nat Commun. 2026 Aug 21;17:8768. doi: 10.1038/s41467-026-76822-8 (PMC13493939; doi:10.1038/s41467-026-76822-8)
Supplement: Supplementary file 2 [file 41467_2026_76822_MOESM1_ESM.pdf]

# A Systematic Review and Meta-Analysis on Heat Thresholds for Maternal Health and Birth Outcomes

*Descriptive Statistics* ........ *2*

**Supplementary Table 1. All included studies in this review (n=103) are classified into year of publication.** ........ **2**

**Supplementary Table 2. All included studies in this review (n=103) are classified into continents in which data was collected.** ........ **2**

**Supplementary Table 3. All included studies in this review (n=103) are classified into countries in which data was collected.** ........ **2**

**Supplementary Table 4. All included studies in this review (n=103) are classified into health outcomes assessed.** ........ **4**

**Supplementary Table 5. All included studies in this review (n=103) are classified into heat indicators used.** ........ **4**

**Supplementary Table 6. All included studies in this review (n=103) are classified into sample sizes.** ........ **5**

**Supplementary Table 7. All included studies in this review (n=103) are classified into study design following the categories used by the OHAT risk of bias assessment tool.** ........ **5**

*Consortium: HIGH Horizon Study Group* ........ *6*

*Supplementary Note 1: PRISMA 2020 abstract checklist* ........ *7*

*Supplementary Note 2: PRISMA 2020 checklist* ........ *8*

## Descriptive Statistics

**Supplementary Table 1. All included studies in this review (n=103) are classified into year of publication.**

| Years | Number of studies | Percentage |
|---|---|---|
| 2025 - 2026 | 11 | 10.68 |
| 2020 - 2024 | 51 | 49.51 |
| 2015 - 2020 | 28 | 27.18 |
| 2010 - 2015 | 9 | 8.73 |
| 2005 - 2010 | 2 | 1.94 |
| 2000 - 2005 | 1 | 0.97 |
| Pre 2000 | 1 | 0.97 |
| TOTAL | 103 | 100 |

**Supplementary Table 2. All included studies in this review (n=103) are classified into continents in which data was collected.**

| Continents | Number of studies | Percentage |
|---|---|---|
| Africa | 4 | 3.88 |
| Asia | 42 | 40.78 |
| Europe | 18 | 17.48 |
| North America | 29 | 28.16 |
| Oceania | 8 | 7.77 |
| South America | 1 | 0.97 |
| Multiple continents | 1 | 0.97 |
| TOTAL | 103 | 100 |

**Supplementary Table 3. All included studies in this review (n=103) are classified into countries in which data was collected.**

| Countries | Number of studies | Percentage |
|---|---|---|
| USA | 24 | 23.30 |
| China | 24 | 23.30 |
| Australia | 8 | 7.77 |
| Canada | 5 | 4.85 |
| Spain | 5 | 4.85 |
| Iran | 4 | 3.88 |
| Italy | 4 | 3.88 |
| France | 3 | 2.91 |
| Israel | 3 | 2.91 |
| Nepal | 3 | 2.91 |
| Belgium | 2 | 1.94 |
| Benin | 2 | 1.94 |
| India | 2 | 1.94 |

| | | |
|---|---|---|
| Japan | 2 | 1.94 |
| Malawi | 2 | 1.94 |
| Nigeria | 2 | 1.94 |
| Pakistan | 2 | 1.94 |
| Uganda | 2 | 1.94 |
| South Africa | 2 | 1.94 |
| Sweden | 2 | 1.94 |
| Zimbabwe | 2 | 1.94 |
| Cyprus | 1 | 0.97 |
| The Gambia | 1 | 0.97 |
| Peru | 1 | 0.97 |
| Angola | 1 | 0.97 |
| Turkey | 1 | 0.97 |
| Korea | 1 | 0.97 |
| Romania | 1 | 0.97 |
| The Netherlands | 1 | 0.97 |
| Northern Ireland | 1 | 0.97 |
| Burkina Faso | 1 | 0.97 |
| Burundi | 1 | 0.97 |
| Ethiopia | 1 | 0.97 |
| Greece | 1 | 0.97 |
| Guinea | 1 | 0.97 |
| Haiti | 1 | 0.97 |
| Kenya | 1 | 0.97 |
| Lesotho | 1 | 0.97 |
| Liberia | 1 | 0.97 |
| Madagascar | 1 | 0.97 |
| Mali | 1 | 0.97 |
| Philippines | 1 | 0.97 |
| Rwanda | 1 | 0.97 |
| Senegal | 1 | 0.97 |
| Sierra Leona | 1 | 0.97 |
| Taiwan | 1 | 0.97 |
| Tajikistan | 1 | 0.97 |
| Timor-Leste | 1 | 0.97 |
| Zambia | 1 | 0.97 |
| TOTAL | 133 | 100 |

"Multiple" indicates that study has collected data from several continents (McElroy et al., 2022)
Only 4 studies were conducted in low-income countries (defined by World Bank Atlas Method of the fiscal year 2024-2025), all of which were conducted in 2022 or thereafter.

- The Gambia; **Bonell** et al., 2023
- Burundi, Ethiopia, Malawi, Uganda; **McElroy** et al., 2022
- Benin; **Tanou** et al. 2022
- Malawi, Mali, Nigeria, Zimbabwe, Madagascar, Senegal, Kenya, Uganda, Lesotho, Burkina Faso, Guinea, Rwanda, Zambia, Sierra Leone, Liberia; **Davenport** et al., 2020

**Supplementary Table 4. All included studies in this review (n=103) are classified into health outcomes assessed.**

| Health Outcomes | Number of studies | Percentage |
|---|---|---|
| Preterm Birth | 54 | 52.42 |
| Low Birth Weight | 20 | 19.42 |
| Stillbirth | 13 | 12.62 |
| Congenital anomalies / heart defects | 8 | 7.77 |
| Preeclampsia / gestational hypertension* | 7 | 6.80 |
| Adverse Pregnancy Outcomes, Adverse Birth Outcomes (composite) | 5 | 4.85 |
| Spontaneous abortions / miscarriage | 6 | 5.83 |
| Premature rupture of membranes (PROM)* | 3 | 2.91 |
| Small for gestational age (SMA) | 4 | 3.88 |
| Umbilical artery resistance index (RI)* | 2 | 1.94 |
| Placenta volume* | 1 | 0.97 |
| Neural tube defects* | 2 | 1.94 |
| Gestational diabetes* | 5 | 4.85 |
| HCGAZ, head circumference-for-age Z-score | 1 | 0.97 |
| Severe Maternal Morbidity* | 1 | 0.97 |
| Placenta weight* | 1 | 0.97 |
| Placenta to birthweight ratio (PFR)* | 1 | 0.97 |
| Placental abruption* | 1 | 0.97 |
| Sleep* | 1 | 0.97 |
| TOTAL | 138 | 100 |

* Maternal Health outcomes (n=25/138, 18.12%)
Note: different total number since some studies have focused on several health outcomes
Percentage is calculated out of the included studies, n=103

**Supplementary Table 5. All included studies in this review (n=103) are classified into heat indicators used.**

| Heat Indicators | Number of studies | Percentage |
|---|---|---|
| Air temperature | 80 | 77.67 |
| AT | 18 | 15.53 |
| Heat Index | 7 | 6.80 |
| WBGT | 5 | 4.85 |
| UTCI | 3 | 2.91 |
| heat variability | 7 | 6.80 |
| TOTAL | 117 | 100 |

NOTE: different total number since some studies have focused on several health outcomes and used several heat indicators. Percentage calculated of total included studies n=103.

**Supplementary Table 6. All included studies in this review (n=103) are classified into sample sizes.**

| Countries | Number of studies | Percentage |
|---|---|---|
| <1000 | 5 | 4.85 |
| 1000-100000 | 50 | 48.54 |
| 100000-1000000 | 37 | 35.92 |
| >1000000 | 11 | 10.68 |
| TOTAL | 103 | 100 |

**Supplementary Table 7. All included studies in this review (n=103) are classified into study design following the categories used by the OHAT risk of bias assessment tool.**

| Study design | Number of studies | Percentage |
|---|---|---|
| Cohort | 62 | 60.19 |
| Case Control | 23 | 22.33 |
| Cross Sectional | 17 | 16.50 |
| Human Controlled Trial | 1 | 0.97 |
| TOTAL | 103 | 100 |

# Consortium: HIGH Horizon Study Group

Stanley Luchters[1,2,3], Matthew Chersich[4], Gloria Maimela[4], Celeste Madondo[4], Shobna Sawry[4], Mags Beksinska[4], Lebohang Radebe[4], Darshnika Lakhoo[4], Ijeoma Solarin[4], Craig Parker[4], Pascalia Munyewende[4], Jean Le Roux[4], Rose Lamola[4], Sibusiso Mkwananzi[4], Nicholas Brink[4], Chuansi Gao[5], Jakob Eggeling[5], Günter Alce[5], Clara Heil[5,] Olof Stephansson[6], Claudia Hanson[6], Nathalie Roos[6], Jeroen de Bont[6], Veronika Tirado[6], Mian Bao[6], Massimo Stafoggia[6,7], Federica Nobile[6,7], Anayda Portela[8] Jørn Toftum[9], Ilona Otto[10], Chloe Brimicombe[10], Katharine Wieser[10], Tobias Monthaler[10], Zeenat Sulaiman[11], Sohail Baloch[11], Aquinius Mung'atia[11], Fortunate Machingura[12], Jetina Tsvaki[12], Thabani Moronzie[12], Concilia Mutasa[12], Brian Mqondisi Sibanda[12], Tariro Chinozvina[12], Elizabeth Dangaiso[12], Jaspar Maguma[12], Bongani Mutimutema[12], Progress Chiworeka[12], Leslie Nyoni[12], Llyod Pisa[12], Jeffrey Dirawo[12], Tapiwa Nyakabau[12], Perkins Watambwa[12], Debra Jackson[13], Veronique Filippi[13], Giulia Greco[13], Nasser Fardousi[13], Olimpia Lamberti[13], Isabelle Lange[13], Giorgia Gon[13], Jo Borghi[13], Paul Lokubal[13], Cherie Part[13], Christos Hadjichristodoulou[14], Barbara Mouchtouri[14], Maria Kyritsi[14], Michalis Koureas[14], Fani Kalala[14], Chara Bogogiannidou[14], Ioanna Voulgaridi[14], Ioannis Messinis[14], Christina Messini[14], Xara Skentou[14], Matthaios Speletas[14], Andreas Flouris[14], Vana Papaevagelou[14], George Daskalakis[14], Androniki Naska[14], Vassiliki Benetou[14], Anastasia (Tania) Konstantinidou[14], Boris Kingma[15], Koen van der Sanden[15], Birgit Kerstens[16]

1 Department of Public Health and Primary Care, Ghent University, Ghent, Belgium
2 Centre for Sexual Health and HIV/AIDS Research, Harare, Zimbabwe
3 Liverpool School of Tropical medicine (LSTM), UK
4 Wits Planetary Health, Faculty of Health Sciences, University of Witwatersrand, Johannesburg, South Africa
5 Faculty of Engineering, Department of Design Sciences, Lund University, Lund, Sweden
6 Karolinska Institutet, Stockholm, Sweden
7 Department of Epidemiology, ASL Roma, Roma, Italy
8 World Health Organization, Geneva, Switzerland
9 Technical University of Denmark, Lyngby, Denmark
10 Wegener Center for Climate and Global Change, University of Graz, Graz, Austria
11 Aga Khan Health Services Kenya, Nairobi, Kenya
12 Centre for Sexual Health and HIV/AIDS Research, Harare, Zimbabwe
13 London School of Hygiene and Tropical Medicine, London, United Kingdom
14 Laboratory of Hygiene and Epidemiology, Medical Faculty, University of Thessaly, Larissa, Greece
15 TNO, Soesterberg, the Netherlands
16 Ghent University, Ghent, Belgium

## Supplementary Note 1: PRISMA 2020 abstract checklist

| Section and Topic | Item # | Checklist item | Reported (Yes/No) |
|---|---|---|---|
| **TITLE** | | | |
| Title | 1 | Identify the report as a systematic review. | Yes |
| **BACKGROUND** | | | |
| Objectives | 2 | Provide an explicit statement of the main objective(s) or question(s) the review addresses. | Yes |
| **METHODS** | | | |
| Eligibility criteria | 3 | Specify the inclusion and exclusion criteria for the review. | Yes |
| Information sources | 4 | Specify the information sources (e.g. databases, registers) used to identify studies and the date when each was last searched. | Yes |
| Risk of bias | 5 | Specify the methods used to assess risk of bias in the included studies. | Yes |
| Synthesis of results | 6 | Specify the methods used to present and synthesise results. | Yes |
| **RESULTS** | | | |
| Included studies | 7 | Give the total number of included studies and participants and summarise relevant characteristics of studies. | Yes |
| Synthesis of results | 8 | Present results for main outcomes, preferably indicating the number of included studies and participants for each. If meta-analysis was done, report the summary estimate and confidence/credible interval. If comparing groups, indicate the direction of the effect (i.e. which group is favoured). | Yes |
| **DISCUSSION** | | | |
| Limitations of evidence | 9 | Provide a brief summary of the limitations of the evidence included in the review (e.g. study risk of bias, inconsistency and imprecision). | Yes |
| Interpretation | 10 | Provide a general interpretation of the results and important implications. | Yes |
| **OTHER** | | | |
| Funding | 11 | Specify the primary source of funding for the review. | Yes |
| Registration | 12 | Provide the register name and registration number. | Yes |

*From:* Page MJ, McKenzie JE, Bossuyt PM, Boutron I, Hoffmann TC, Mulrow CD, et al. The PRISMA 2020 statement: an updated guideline for reporting systematic

# Supplementary Note 2: PRISMA 2020 checklist

| Section and Topic | Item # | Checklist item | Location where item is reported |
|---|---|---|---|
| **TITLE** | | | |
| Title | 1 | Identify the report as a systematic review. | p. 1 |
| **ABSTRACT** | | | |
| Abstract | 2 | See the PRISMA 2020 for Abstracts checklist. | p. 1 (also additional file) |
| **INTRODUCTION** | | | |
| Rationale | 3 | Describe the rationale for the review in the context of existing knowledge. | p. 3 |
| Objectives | 4 | Provide an explicit statement of the objective(s) or question(s) the review addresses. | p. 3 |
| **METHODS** | | | |
| Eligibility criteria | 5 | Specify the inclusion and exclusion criteria for the review and how studies were grouped for the syntheses. | p. 13 |
| Information sources | 6 | Specify all databases, registers, websites, organisations, reference lists and other sources searched or consulted to identify studies. Specify the date when each source was last searched or consulted. | p. 14 |
| Search strategy | 7 | Present the full search strategies for all databases, registers and websites, including any filters and limits used. | p. 14-15, Appendix 1 |
| Selection process | 8 | Specify the methods used to decide whether a study met the inclusion criteria of the review, including how many reviewers screened each record and each report retrieved, whether they worked independently, and if applicable, details of automation tools used in the process. | p. 15 |
| Data collection process | 9 | Specify the methods used to collect data from reports, including how many reviewers collected data from each report, whether they worked independently, any processes for obtaining or confirming data from study investigators, and if applicable, details of automation tools used in the process. | p. 15 |
| Data items | 10a | List and define all outcomes for which data were sought. Specify whether all results that were compatible with each outcome domain in each study were sought (e.g. for all measures, time points, analyses), and if not, the methods used to decide which results to collect. | p. 15-16 |
| | 10b | List and define all other variables for which data were sought (e.g. participant and intervention characteristics, funding sources). Describe any assumptions made about any missing or unclear information. | p. 15-16 |
| Study risk of | 11 | Specify the methods used to assess risk of bias in the included studies, | p. 16 |

| Section and Topic | Item # | Checklist item | Location where item is reported |
|---|---|---|---|
| bias assessment | | including details of the tool(s) used, how many reviewers assessed each study and whether they worked independently, and if applicable, details of automation tools used in the process. | |
| Effect measures | 12 | Specify for each outcome the effect measure(s) (e.g. risk ratio, mean difference) used in the synthesis or presentation of results. | p. 16 |
| Synthesis methods | 13a | Describe the processes used to decide which studies were eligible for each synthesis (e.g. tabulating the study intervention characteristics and comparing against the planned groups for each synthesis (item #5)). | p. 16 |
| | 13b | Describe any methods required to prepare the data for presentation or synthesis, such as handling of missing summary statistics, or data conversions. | p. 15 |
| | 13c | Describe any methods used to tabulate or visually display results of individual studies and syntheses. | p. 17 |
| | 13d | Describe any methods used to synthesize results and provide a rationale for the choice(s). If meta-analysis was performed, describe the model(s), method(s) to identify the presence and extent of statistical heterogeneity, and software package(s) used. | p. 17 |
| | 13e | Describe any methods used to explore possible causes of heterogeneity among study results (e.g. subgroup analysis, meta-regression). | p. 16 |
| | 13f | Describe any sensitivity analyses conducted to assess robustness of the synthesized results. | p.16 |
| Reporting bias assessment | 14 | Describe any methods used to assess risk of bias due to missing results in a synthesis (arising from reporting biases). | p. 15 |
| Certainty assessment | 15 | Describe any methods used to assess certainty (or confidence) in the body of evidence for an outcome. | p. 16 |
| **RESULTS** | | | |
| Study selection | 16a | Describe the results of the search and selection process, from the number of records identified in the search to the number of studies included in the review, ideally using a flow diagram. | p. 4 |
| | 16b | Cite studies that might appear to meet the inclusion criteria, but which were excluded, and explain why they were excluded. | p. 5 |
| Study characteristics | 17 | Cite each included study and present its characteristics. | Supplementary Data 1 |
| Risk of bias in studies | 18 | Present assessments of risk of bias for each included study. | Supplementary Data 3 |
| Results of | 19 | For all outcomes, present, for each study: (a) summary statistics for each group | Supplementary Data 1 |

| Section and Topic | Item # | Checklist item | Location where item is reported |
|---|---|---|---|
| individual studies | | (where appropriate) and (b) an effect estimate and its precision (e.g. confidence/credible interval), ideally using structured tables or plots. | |
| Results of syntheses | 20a | For each synthesis, briefly summarise the characteristics and risk of bias among contributing studies. | p. 4-8 |
| | 20b | Present results of all statistical syntheses conducted. If meta-analysis was done, present for each the summary estimate and its precision (e.g. confidence/credible interval) and measures of statistical heterogeneity. If comparing groups, describe the direction of the effect. | p. 5 |
| | 20c | Present results of all investigations of possible causes of heterogeneity among study results. | p. 6-8 |
| | 20d | Present results of all sensitivity analyses conducted to assess the robustness of the synthesized results. | p. 5-6 |
| Reporting biases | 21 | Present assessments of risk of bias due to missing results (arising from reporting biases) for each synthesis assessed. | p. 8-9 |
| Certainty of evidence | 22 | Present assessments of certainty (or confidence) in the body of evidence for each outcome assessed. | p. 6-8 |
| **DISCUSSION** | | | |
| Discussion | 23a | Provide a general interpretation of the results in the context of other evidence. | p. 9-10 |
| | 23b | Discuss any limitations of the evidence included in the review. | p. 12 |
| | 23c | Discuss any limitations of the review processes used. | p. 11-12 |
| | 23d | Discuss implications of the results for practice, policy, and future research. | p. 10-11 |
| **OTHER INFORMATION** | | | |
| Registration and protocol | 24a | Provide registration information for the review, including register name and registration number, or state that the review was not registered. | p. 1, p. 14 |
| | 24b | Indicate where the review protocol can be accessed, or state that a protocol was not prepared. | p. 14 |
| | 24c | Describe and explain any amendments to information provided at registration or in the protocol. | p. 14 |
| Support | 25 | Describe sources of financial or non-financial support for the review, and the role of the funders or sponsors in the review. | p. 25 |
| Competing interests | 26 | Declare any competing interests of review authors. | p. 25 |
| Availability of data, code and other | 27 | Report which of the following are publicly available and where they can be found: template data collection forms; data extracted from included studies; data used for all analyses; analytic code; any other materials used in the review. | Supplementary Data 1 & 2 for data extracted from all studies<br>Figshare: |

| Section and Topic | Item # | Checklist item | Location where item is reported |
|---|---|---|---|
| materials | | | https://doi.org/10.6084/m9.figshare.32802512 for data used from included meta-analysis studies |

*From:* Page MJ, McKenzie JE, Bossuyt PM, Boutron I, Hoffmann TC, Mulrow CD, et al. The PRISMA 2020 statement: an updated guideline for reporting systematic reviews. BMJ 2021;372:n71. doi: 10.1136/bmj.n71. This work is licensed under CC BY 4.0. To view a copy of this license, visit https://creativecommons.org/licenses/by/4.0/
