# Supplementary material for: A systematic review and meta-analysis on heat thresholds for maternal health and birth outcomes
Source: Nat Commun. 2026 Aug 21;17:8768. doi: 10.1038/s41467-026-76822-8 (PMC13493939; doi:10.1038/s41467-026-76822-8)
Supplement: Supplementary file 3 [file 41467_2026_76822_MOESM2_ESM.pdf]

Description of Additional Supplementary Files

Supplementary Data 1. Characteristics of studies included

Supplementary Data 2. Assessment of covariate use

Supplementary Data 3. OHAT risk of bias assessment

Supplementary Data 4. Leave-one-study-out (LOSO) sensitivity analysis

Supplementary Data 5. Baseline risk sensitivity analysis
