# Supplementary material for: A systematic review and meta-analysis on heat thresholds for maternal health and birth outcomes
Source: Nat Commun. 2026 Aug 21;17:8768. doi: 10.1038/s41467-026-76822-8 (PMC13493939; doi:10.1038/s41467-026-76822-8)
Supplement: Supplementary file 4 [file 41467_2026_76822_MOESM8_ESM.pdf]

nature portfolio

Corresponding author(s): Clara Sofia Heil

Last updated by author(s): Jun 26, 2026



# Reporting Summary

Nature Portfolio wishes to improve the reproducibility of the work that we publish. This form provides structure for consistency and transparency in reporting. For further information on Nature Portfolio policies, see our Editorial Policies and the Editorial Policy Checklist.

## Statistics

For all statistical analyses, confirm that the following items are present in the figure legend, table legend, main text, or Methods section.

| n/a | Confirmed | |
|---|---|---|
| ☐ | ☒ | The exact sample size ($n$) for each experimental group/condition, given as a discrete number and unit of measurement |
| ☒ | ☐ | A statement on whether measurements were taken from distinct samples or whether the same sample was measured repeatedly |
| ☐ | ☒ | The statistical test(s) used AND whether they are one- or two-sided<br>*Only common tests should be described solely by name; describe more complex techniques in the Methods section.* |
| ☐ | ☒ | A description of all covariates tested |
| ☐ | ☒ | A description of any assumptions or corrections, such as tests of normality and adjustment for multiple comparisons |
| ☐ | ☒ | A full description of the statistical parameters including central tendency (e.g. means) or other basic estimates (e.g. regression coefficient) AND variation (e.g. standard deviation) or associated estimates of uncertainty (e.g. confidence intervals) |
| ☒ | ☐ | For null hypothesis testing, the test statistic (e.g. $F$, $t$, $r$) with confidence intervals, effect sizes, degrees of freedom and $P$ value noted<br>*Give P values as exact values whenever suitable.* |
| ☒ | ☐ | For Bayesian analysis, information on the choice of priors and Markov chain Monte Carlo settings |
| ☒ | ☐ | For hierarchical and complex designs, identification of the appropriate level for tests and full reporting of outcomes |
| ☒ | ☐ | Estimates of effect sizes (e.g. Cohen's $d$, Pearson's $r$), indicating how they were calculated |

*Our web collection on statistics for biologists contains articles on many of the points above.*

## Software and code

Policy information about availability of computer code

| | |
|---|---|
| Data collection | EndNote25 used for reference management, Covidence for screening, in- and exclusion sorting and structuring, WebPlotDigitizer 5.2 for non-numeric data collection. |
| Data analysis | Rstudio, Version 1.3.1093, PBC, Boston, Massachusetts, United States, package “metafor”. |

For manuscripts utilizing custom algorithms or software that are central to the research but not yet described in published literature, software must be made available to editors and reviewers. We strongly encourage code deposition in a community repository (e.g. GitHub). See the Nature Portfolio guidelines for submitting code & software for further information.

## Data

Policy information about availability of data

All manuscripts must include a data availability statement. This statement should provide the following information, where applicable:

- Accession codes, unique identifiers, or web links for publicly available datasets
- A description of any restrictions on data availability
- For clinical datasets or third party data, please ensure that the statement adheres to our policy

Source data are provided with this paper. Data extracted from previously published studies are available in Supplementary Data 1-2. The raw data underlying the Tmax meta-regressions and the corresponding analysis outputs have been deposited in Figshare repository under accession code: https://doi.org/10.6084/

m9.figshare.3280251297. Additional study characteristics, risk-of-bias assessments, and sensitivity analyses are provided in the Supplementary Information and Supplementary Data files. There was no custom code developed for the study analyses.

## Research involving human participants, their data, or biological material

Policy information about studies with human participants or human data. See also policy information about sex, gender (identity/presentation), and sexual orientation and race, ethnicity and racism.

| | |
|---|---|
| Reporting on sex and gender | Reported according to sex and gender reporting: SAGER (Sex and Gender Equity in Research) guidelines. Captured in Abstract and Methods. Participants within studies included in the review were limited to individuals capable of becoming pregnant, including women and those identifying differently. No disaggregated sex or gender analysis was therefore possible. Information on gender generally not the focus and unavailable in the included studies, precluding gender-specific analyses. |
| Reporting on race, ethnicity, or other socially relevant groupings | No race and ethnicity was reported in this study.<br>The geographical location and World Bank definitions for country level incomes was used as descriptive statistics. |
| Population characteristics | *Describe the covariate-relevant population characteristics of the human research participants (e.g. age, genotypic information, past and current diagnosis and treatment categories). If you filled out the behavioural & social sciences study design questions and have nothing to add here, write "See above."* |
| Recruitment | *Describe how participants were recruited. Outline any potential self-selection bias or other biases that may be present and how these are likely to impact results.* |
| Ethics oversight | *Identify the organization(s) that approved the study protocol.* |

Note that full information on the approval of the study protocol must also be provided in the manuscript.

# Field-specific reporting

Please select the one below that is the best fit for your research. If you are not sure, read the appropriate sections before making your selection.

☐ Life sciences ☐ Behavioural & social sciences ☒ Ecological, evolutionary & environmental sciences

For a reference copy of the document with all sections, see nature.com/documents/nr-reporting-summary-flat.pdf

# Ecological, evolutionary & environmental sciences study design

All studies must disclose on these points even when the disclosure is negative.

| | |
|---|---|
| Study description | We conducted a systematic literature review following the 2020 Preferred Reporting Items for Systematic Reviews and Meta-Analysis (PRISMA) reporting guidelines and the Guidelines for Meta-Analyses and Systematic Reviews of Observational Studies (MOOSE). We synthesised data on heat indicators used, health outcomes assessed and carried out meta-analyses to determine heat-health thresholds for pregnant individuals. |
| Research sample | We included all peer-reviewed articles reporting heat health thresholds linked to adverse maternal health and birth outcomes. Included were all study designs, except for systematic review and editorials and commentaries. We also excluded grey literature. |
| Sampling strategy | A systematic search was carried out across five databases MEDLINE (PubMed), Web of Science, Scopus, GREENFile and Cinahl without restrictions on date nor geographical location. Title and abstract screening was performed by a single person, full-text screening involved two independent reviewers. Data extraction was carried out by a single reviewer after piloting an extraction framework with two other co-authors. Extracted data was thereafter checked in full by an independent co-author. |
| Data collection | We extracted data on year of publication, aim, study design, location, climate zone, data collection period, population source, exposure definition, heat information, outcome, main finding, maternal age, smoking, BMI, air pollution, parity, SES, cooling behaviour, gestational period. |
| Timing and spatial scale | The systematic search with its search string used across the five databases included literature until June 2024. Forward citation chasing until March 2026 was conducted in addition. No geographical limitations were imposed. |
| Data exclusions | The exact exclusion criteria are included in the manuscript. |
| Reproducibility | The full search string and strategy can be found in the manuscript to identify the exact steps taken and reproduce the results reported. |
| Randomization | *Describe how samples/organisms/participants were allocated into groups. If allocation was not random, describe how covariates were controlled. If this is not relevant to your study, explain why.* |
| Blinding | *Describe the extent of blinding used during data acquisition and analysis. If blinding was not possible, describe why OR explain why blinding was not relevant to your study.* |

Did the study involve field work? ☐ Yes ☒ No

# Reporting for specific materials, systems and methods

We require information from authors about some types of materials, experimental systems and methods used in many studies. Here, indicate whether each material, system or method listed is relevant to your study. If you are not sure if a list item applies to your research, read the appropriate section before selecting a response.

## Materials & experimental systems

| n/a | Involved in the study |
|---|---|
| ☒ | ☐ Antibodies |
| ☒ | ☐ Eukaryotic cell lines |
| ☒ | ☐ Palaeontology and archaeology |
| ☒ | ☐ Animals and other organisms |
| ☒ | ☐ Clinical data |
| ☒ | ☐ Dual use research of concern |
| ☒ | ☐ Plants |

## Methods

| n/a | Involved in the study |
|---|---|
| ☒ | ☐ ChIP-seq |
| ☒ | ☐ Flow cytometry |
| ☒ | ☐ MRI-based neuroimaging |

## Plants

| | |
|---|---|
| Seed stocks | *Report on the source of all seed stocks or other plant material used. If applicable, state the seed stock centre and catalogue number. If plant specimens were collected from the field, describe the collection location, date and sampling procedures.* |
| Novel plant genotypes | *Describe the methods by which all novel plant genotypes were produced. This includes those generated by transgenic approaches, gene editing, chemical/radiation-based mutagenesis and hybridization. For transgenic lines, describe the transformation method, the number of independent lines analyzed and the generation upon which experiments were performed. For gene-edited lines, describe the editor used, the endogenous sequence targeted for editing, the targeting guide RNA sequence (if applicable) and how the editor was applied.* |
| Authentication | *Describe any authentication procedures for each seed stock used or novel genotype generated. Describe any experiments used to assess the effect of a mutation and, where applicable, how potential secondary effects (e.g. second site T-DNA insertions, mosiacism, off-target gene editing) were examined.* |
