# Supplementary material for: A systematic review and meta-analysis on heat thresholds for maternal health and birth outcomes
Source: Nat Commun. 2026 Aug 21;17:8768. doi: 10.1038/s41467-026-76822-8 (PMC13493939; doi:10.1038/s41467-026-76822-8)
Supplement: Supplementary file 5 [file 41467_2026_76822_MOESM9_ESM.pdf]

nature portfolio

Peer Review File

A Systematic Review and Meta-Analysis on Heat Thresholds for Maternal Health and Birth Outcomes

Corresponding Author: Ms Clara Heil

This file contains all reviewer reports in order by version, followed by all author rebuttals in order by version.

Version 0:

Reviewer comments:

Reviewer #1

(Remarks to the Author)
This is an incredibly exciting and important research topic, and I'm glad the authors have undertaken this work. The synthesis of evidence on heat thresholds and pregnancy outcomes is both timely and urgently needed in the context of accelerating climate change and the disproportionate health impacts that many women and girls face. This review has the potential to make a valuable contribution to the evidence base informing early warning systems. However, I do have several major and minor concerns that I believe warrant further attention, listed below on a point-to-point basis.

Introduction
Lines 31-68 - The flow of the introduction can be improved by following a clearer background -> problem -> research gap -> aim structure. For example, following something like: climate change as a health threat, its impact on maternal and birth outcomes, importance of heat thresholds, lack of standardised definitions/thresholds for maternal and birth outcomes, aim of this review. Which would translate in:
Paragraph 1: Broad context and relevance (climate as a health threat, its impact on maternal and birth outcomes)
Paragraph 2: Challenge of defining heat threshold, and the need to do so
Paragraph 3: Existing literature, literature gap, need for this work
Paragraph 4: Aims and significance of this work.

Lines 40-41 – While the identification of heat thresholds is mostly discussed in the context of early warning systems, they also have broader applications — including informing clinical care guidelines, occupational safety regulations, urban planning standards, maternal adaptation policies, and health equity strategies in the face of rising temperatures. The authors may consider mentioning this as well.

At the authors' discretion, it may be helpful to include a brief statement on the broader impacts of climate system changes on pregnancy outcomes, as well as any gender-related differences in exposure, risk, or vulnerability to climate-related health impacts. This could help situate the review within a wider discussion of gendered health vulnerabilities in the context of climate change.

Methods / results
- The timing of heat exposure during pregnancy may differentially affect maternal and birth outcomes. For instance, third-trimester exposure has been particularly associated with preterm birth, whereas first-trimester exposure may be more relevant for congenital anomalies. However, it is unclear whether the authors accounted for the timing of heat exposure in their meta-analyses (i.e., whether exposures across different trimesters were combined). Clarifying and contextualising the timing of exposure in the results would enhance the interpretation and help assess potential sources of heterogeneity across studies. If data permit, it would be valuable to disaggregate the analyses by timing of exposure.
- It is unclear from the description of the data extraction and meta-analyses whether the effect estimates included from individual studies were consistently those from the most fully adjusted models, crude estimates, or based on another selection criterion. This should be clarified. If possible, it may be worth conducting sensitivity analyses with the crude models.
- The authors mention that they "excluded effect estimates that were based on various lag days." Does this mean that only effect estimates based on lag 0 were included? This should be clarified, as it is well established that different lag structures

can influence the observed association between heat exposure and health outcomes. Providing a rationale for the choice of lag structure—or specifying how lag-related heterogeneity was handled would be helpful.
- Heat thresholds are known to vary according to individual and environmental factors, such as age, acclimatisation, activity level, humidity, pre-existing health conditions, and access to cooling. These important sources of variation do not appear to be accounted for in the meta-analyses or discussed in the interpretation of results. If individual study effect estimates are disaggregated by population subgroups, it may be valuable to explore sensitivity analyses. Similarly, examining differences across climate zones could offer further insight (the authors do divide study locations into tropical, arid temperatre and continental in line 78-79). For instance, individuals living in Australia are likely to be more physiologically adapted to heat than those in the UK, and therefore may have higher thresholds for adverse health effects. Acknowledging and, if possible, analysing these differences would strengthen the manuscript.
- The meta-analyses presented in Figure 3 and described in the Methods appear to combine different maternal and birth outcomes. I have some concerns about the validity of this approach, as different outcomes may have distinct heat thresholds and may be differentially influenced by the timing of exposure during pregnancy. Pooling such heterogeneous outcomes could obscure meaningful differences and reduce the interpretability of the findings. It would be helpful if the authors could justify this approach and, if possible, provide disaggregated analyses by outcome type or by maternal OR neonatal outcomes.
- It would be helpful if the authors included a brief section with key definitions at the beginning of the Methods, clearly specifying how they define “heat threshold” (i.e. what qualifies as a heat exposure for inclusion) and “adverse maternal health and birth outcomes.” This would improve clarity and consistency for the reader regarding inclusion criteria. Currently, additional information on the definition of heat and maternal/neonatal health appears later in the “Data analysis” section (line 563 onwards), which may be confusing without earlier clarification.
Supplementary appendix Table S1 – Is it correct that, across all included studies, only smoking, BMI, and air pollution were assessed as covariates? Or are these simply the only covariates for which data were extracted? I ask because I would have expected a broader range of confounders to be reported in the original studies, such as maternal age, socioeconomic status, parity, or pre-existing health conditions. It would be great if this can be clarified.
Supplementary Table S2 – The table currently includes an extensive amount of citation information (e.g., full title, DOI, and author list), which is not essential for a study characteristics and results table. Instead, this space could be used more effectively to present key details on individual studies and results. I recommend consolidating the citation information into a single “Study” column (e.g., “Tiwari et al. 2024”) and expanding the table to include relevant methodological and outcome-related information such as population source, time period of data collection, exposure definition + acertainment, outcome(s) definition + ascertainment, covariates adjusted for, and main findings. This would enhance the utility of the table, and is particularly relevant for those studies that were not included in the meta-analyses given their results are only very limitedly discussed. The authors can also consider splitting the table up in a study characteristics and a results table.
Table 1 – To improve clarity, consider subdividing the “Health outcomes” section into maternal and neonatal outcomes. This would help distinguish which outcomes directly affect the mother versus the child.
Line 100-102 – The authors mention “additional environmental parameters included,” but it is unclear whether this means that temperature variables were jointly assessed with these parameters (e.g., in multi-variable models), or whether an individual effect estimate for temperature was still reported. Clarifying this distinction would help interpret how temperature-specific effects were isolated and included in the meta-analyses.
Summary of effects section – This section could benefit from a clearer explanation. It would be helpful to understand why such a large number of effect estimates were extracted. Are these based on different model adjustments, lag times, exposure time windows, temperature definitions (e.g., Tmax, Tmin, mean temperature), or other variations within individual studies? Providing this detail would clarify the heterogeneity of the extracted estimates and how they were handled in the analysis.
Line 502-503 – In addition to referencing the PRISMA checklist, it would be appropriate to also include the MOOSE guidelines for reporting meta-analyses of observational studies: https://jamanetwork.com/journals/jamasurgery/article-abstract/2778476
Line 506 – The authors state that all study designs were included; however, if observed correctly, no ecological studies appear to have been included. Were these simply not identified?
Line 508-109 – Consider moving the sentence “Studies in a language not spoken by any of the authors were also excluded” to the beginning of the Methods section, alongside a list of the languages spoken by the authors. This would improve clarity while avoiding disruption to the flow of the inclusion/exclusion criteria.
Line 513 - Please specify the languages spoken by the authors to clarify the exclusion criterion: “Studies in a language not spoken by any of the authors were also excluded.”
Line 519-520 – The selection of databases searched is likely to bias the results toward English-language journals. This potential language and publication bias should be acknowledged as a limitation in the limitations section.
Appendix 2 – The search strategy does not include specific terms related to individual maternal or birth outcomes. This raises the concern that relevant studies focusing on particular outcomes (e.g., preeclampsia, gestational diabetes, stillbirth, congenital anomalies) may have been missed if they did not also include broader terms such as “pregnant” or “postpartum.” Did the authors test whether incorporating specific outcome-related keywords affected the search yield?
Line 543-544 – It would be helpful to clarify whether the authors conducted double data extraction on a subset of studies to assess inter-rater variability, or whether the data extraction tool was piloted in any way.

Discussion - Much of the discussion is centred around the need to develop early warning systems (EWS) for heat-related pregnancy outcomes, but it does not mention any existing tools or initiatives already in development. For example, the HIGH HORIZONS initiative is currently developing the MotherHeat Alert app, which aims to warn pregnant and postpartum individuals—as well as maternity care providers—about rising heat in their area and provide guidance on how to stay safe. Reflecting on such existing or emerging tools (and what thresholds they are based on) would add depth to the discussion and highlight how research findings like those presented in this review could support or refine these real-world applications.
Several of my comments above raise considerations and potential limitations related to the meta-analyses and the

interpretation of results—such as the timing of heat exposure during pregnancy, the pooling of different maternal and birth outcomes, and variability in heat thresholds across populations and climates, definitions of temperature. These points may be worth elaborating on more fully in the Discussion section, as they could influence both the robustness and generalisability of the findings.

Reviewer #2

(Remarks to the Author)
I read this manuscript with great interest, as it addresses a timely and important public health topic: defining heat exposure thresholds associated with adverse pregnancy and birth outcomes through a systematic review and meta-analysis. The study has clear relevance to maternal health policy, particularly in the context of climate change. However, several methodological concerns require careful attention before the manuscript can be considered acceptable for publication.

The central statistical approach used to derive odds ratios (OR) for adverse pregnancy outcomes relies on the equation: $\textit{Odds Ratio for adverse pregnancy outcomes} = e^{0.39897-0.03937\times Tmax+(-0.00094\times Tmax^2)}$
Currently, the manuscript provides limited details regarding the selection and validation of this model. Essential information about model development, assumption checking, standard errors, residual diagnostics, and confidence intervals around predicted thresholds is missing. Moreover, this model is built on data from only 14 studies (192 data points) without external or sensitivity validation. Greater transparency and comprehensive model diagnostics are necessary to enhance confidence in these critical results.

Additionally, the manuscript defines heat exposure exclusively in terms of daily maximum temperature (Tmax), without considering duration, frequency, or cumulative exposure. Given that prolonged heat exposure and repeated heatwaves are likely to have more significant health implications than single-day exposures, incorporating these dimensions would substantially improve the applicability and real-world relevance of the findings.

The meta-analysis also reveals substantial statistical heterogeneity ($I^2 = 88.2\%$). Although acknowledged, the authors have not sufficiently explored potential sources of this variability. Conducting subgroup analyses or meta-regressions stratified by climate zone, socioeconomic status, or study design would help clarify the reasons for heterogeneity and increase confidence in combining results into global thresholds.

The current conversion of relative risks (RR), hazard ratios (HR), and prevalence ratios (PR) into odds ratios (OR) uniformly assumes a baseline risk of 7.5%. The rationale for this assumption is unclear, and no sensitivity analysis has been performed to test its appropriateness. Given the diverse populations and outcomes included, the authors should explicitly address how this assumption may influence their results.

Key confounding factors such as maternal BMI, smoking, air pollution, and pre-existing health conditions were noted but not adjusted for in the analyses. Incorporating these important covariates into the analytical framework would significantly strengthen the robustness and interpretability of the findings.

Furthermore, the manuscript does not discuss or account for cooling behaviors such as air-conditioning use, shaded environments, or workplace ventilation. Given their substantial influence on individual heat exposure, addressing these factors explicitly is essential for translating findings into practical public health recommendations and early warning systems.

The studies included are heavily weighted toward high and upper middle income countries, with only two studies from low income settings. To enhance global applicability and relevance, greater emphasis should be placed on addressing the geographic imbalance and discussing implications for vulnerable populations in resource-limited settings.

Finally, some relevant studies appear to be omitted, notably Xiong et al. (Nature Communications, 2020) and Mao et al. (Hypertension in Pregnancy, 2023). Including these works would enhance comprehensiveness and reduce potential biases in the synthesis.

Reviewer #3

(Remarks to the Author)
Thank you for asking me to review this interesting systematic review and meta-analysis on heat thresholds for maternal health and birth outcomes. The research covers an important area. However, the reporting does not meet current standards in the field which makes assessing the quality of the methods and therefore the robustness of findings challenging. I have therefore limited by comments to the reporting of the systematic review methods as without confidence in these, the analysis/results do not merit further consideration.

1. PRISMA guidelines are guidelines for reporting of systematic reviews and meta-analyses, they are not guidelines for the conduct of a review. Stating that the review has been conducted according to PRISMA guidelines is therefore incorrect. Moreover, although the authors refer to the guidelines they have not followed them in the reporting of the review. The abstract is missing multiple methodological details e.g. data sources, dates of searches, methods of study selection, data extraction and risk of bias assessment etc. etc. Please refer to the PRISMA guidelines for reporting abstracts.

2. A systematic review can only ever be as good as the search underlying the selection of included studies. I would expect to

see a wider range of databases including some that index environmental journals, and some supplementary search methods e.g. forwards and backwards citation chasing, website searching etc. Also Web of Science is a platform hosting multiple specific databases, it is not correct to say that Web of Science was searched without naming the specific databases that were searched.

3. It is disappointing to see the methods section as an appendix, readers can not judge the robustness of the research without sight of the methods and these should be easily accessible.

4. The methods section is confused, methods for study selection are described in two different places. It is not clear what is meant by Covidence being used for the synthesis and selection of literature - in what way was Covidence used to synthesise?

5. Data extraction was performed by one reviewer and not checked by a second. This is a key limitation. Best practice methods involve two reviewers because errors/differences in interpretation are common in data extraction.

6. Although risk of bias was assessed - no detail is provided about how many reviewers performed this task, how discrepancies were dealt with or what the impact of the findings was on the synthesis.

Version 1:

Reviewer comments:

Reviewer #1

(Remarks to the Author)
Thank you for the revised manuscript. Overall, great work with the revisions! I do have a few more points of clarification and improvement:

- The authors have now clarified that they selected lag0 effect estimates. It should be made much more explicit, ideally in the abstract and early in the methods, that this study focuses specifically on immediate (lag 0) effects on maternal health and birth outcomes. I agree with Reviewer 2 that a major limitation of the meta-analysis is the lack of assessment of duration, frequency, and cumulative exposure, and the restriction to lag 0 effects only. While I appreciate that studies examining duration, frequency, and cumulative exposure may vary in their definitions and reporting, many primary studies typically present results across multiple lag structures or include sensitivity analyses. It would therefore seem feasible to extract these data to conduct additional meta-analyses comparing short-lag and longer-lag effects, or at minimum to provide a clearer justification for restricting the analysis to lag 0 (is it reasonable to assume the effects as this immediate on maternal and neonatal outcomes?). This was also done in the previously published meta-analyses by Lakhoo et al.

- The authors have now clarified that have consistently used the least adjusted models to pool in the meta-analyses “For both the meta-analysis and the narrative analysis, we consistently extracted effect estimates from the least-adjusted models, to minimise discrepancies arising from differences in covariates across adjusted models.” This approach is not ideal, as relying on the least-adjusted models is likely to introduce substantial residual confounding. In general, the conventional approach in meta-analyses of observational studies is to extract estimates from the most fully adjusted (i.e., “best”) model available, on the assumption that the investigators have selected covariates to minimise confounding and provide the most valid effect estimate. I would therefore encourage you to justify this methodological decision more explicitly, and to change the analyses using the most fully adjusted estimates as primary analyses - potentially alongside sensitivity analyses of the crude models. Have analyses using the “best” models been conducted?
- Although I agree with Reviewer 3 that placement of the methods within the main text would have been ideal, the constraints on manuscript length make it appropriate to include the methodological details in the appendix, thereby allowing sufficient space for a thorough discussion of the results. Overall, the clarity of the methodology has improved considerably.

- You mentioned that you excluded studies if they were not published in a language that any of the team members were fluent in. I would generally caution against any exclusion of studies based on language given we now have so many translation tools that would support their inclusion. Were there any relevant studies that were excluded based on language? If so, I would reconsider this and include them.

- Regarding data extraction, you have partially clarified your approach in your response to Reviewer 3. However, it would be helpful to state more explicitly whether all studies were extracted independently by one reviewer and subsequently checked in full by a second reviewer. While dual independent extraction with consensus is the gold standard, it is acceptable for one reviewer to undertake the extraction (following initial piloting), provided that a second reviewer verifies all extracted data. Given that this is a meta-analysis, the accuracy of the extracted data is critical to the validity of the pooled estimates

- It looks like something has gone wrong with the table in the appendix. I recommend to submit this as a horizontal table to make it readable for the reader. At this point, I can’t see which information belongs to which study.

Reviewer #2

(Remarks to the Author)

Reviewer #3

(Remarks to the Author)
Thank you for asking me to consider the author's response to previous peer review comments. Whilst the author team have made some changes to the manuscript in response to comments, in many places they haven't strengthened the methods underlying the review and as such some concerns remain.

For example, they identified an additional five studies eligible for inclusion in the meta-regression after undertaking backward citation chasing. This represents a quarter of the total included studies in this analysis and could suggest that the initial search was not sufficient. It also indicates that further supplementary searching e.g. forward citation chasing may be necessary. Furthermore, the authors suggest that their choice of databases is sufficient - this isn't based on any evidence and with the identification of additional studies through backward citation chasing, may not be correct. I note that one of the other peer reviewers also commented on the limited number of databases used, and questioned the search strategy. Further review of the search strategy by an information specialist with expertise in developing searches for systematic reviews would be advisable.

The author’s approach to checking data extraction is disappointing and may indicate that the synthesis contains errors.

I appreciate the word count restrictions, but the abstract would benefit from inclusion of limitations in the methods used to conduct the review, not only the limitations in the evidence identified. Many of the methodological limitations e.g. limited choice of databases, limited supplementary searching, no systematic checking of data extraction have the potential to undermine confidence in the review findings.

Version 2:

Reviewer comments:

Reviewer #1

(Remarks to the Author)
I thank the authors for carefully addressing the previous feedback and revising the manuscript accordingly. I have no further comments and congratulate them on an important contribution.

Reviewer #3

(Remarks to the Author)
Thank you for the opportunity to review this revised manuscript.

I note that the authors have now dealt thoroughly with the methodological issues raised in previous iterations of the peer review process and the manuscript is improved as a result of these changes.

Open Access This Peer Review File is licensed under a Creative Commons Attribution 4.0 International License, which permits use, sharing, adaptation, distribution and reproduction in any medium or format, as long as you give appropriate credit to the original author(s) and the source, provide a link to the Creative Commons license, and indicate if changes were made.
In cases where reviewers are anonymous, credit should be given to 'Anonymous Referee' and the source.
The images or other third party material in this Peer Review File are included in the article's Creative Commons license, unless indicated otherwise in a credit line to the material. If material is not included in the article's Creative Commons license and your intended use is not permitted by statutory regulation or exceeds the permitted use, you will need to obtain permission directly from the copyright holder.
To view a copy of this license, visit https://creativecommons.org/licenses/by/4.0/

Reviewer comments, point by point

Reviewer #1 (Remarks to the Author):

This is an incredibly exciting and important research topic, and I'm glad the authors have undertaken this work. The synthesis of evidence on heat thresholds and pregnancy outcomes is both timely and urgently needed in the context of accelerating climate change and the disproportionate health impacts that many women and girls face. This review has the potential to make a valuable contribution to the evidence base informing early warning systems. However, I do have several major and minor concerns that I believe warrant further attention, listed below on a point-to-point basis.

Introduction
Lines 31-68 - The flow of the introduction can be improved by following a clearer background -> problem -> research gap -> aim structure. For example, following something like: climate change as a health threat, its impact on maternal and birth outcomes, importance of heat thresholds, lack of standardised definitions/thresholds for maternal and birth outcomes, aim of this review. Which would translate in:
Paragraph 1: Broad context and relevance (climate as a health threat, its impact on maternal and birth outcomes)
Paragraph 2: Challenge of defining heat threshold, and the need to do so
Paragraph 3: Existing literature, literature gap, need for this work
Paragraph 4: Aims and significance of this work.
Thank you for this very constructive comment. We have now changed the introduction structure according to these suggestions (line 22-64) and hope that it has improved the clarity.

Lines 40-41 – While the identification of heat thresholds is mostly discussed in the context of early warning systems, they also have broader applications — including informing clinical care guidelines, occupational safety regulations, urban planning standards, maternal adaptation policies, and health equity strategies in the face of rising temperatures. The authors may consider mentioning this as well.
This is a good point; we have added a sentence listing additional aspects where heat thresholds may have important implications (line 38-40).

At the authors' discretion, it may be helpful to include a brief statement on the broader impacts of climate system changes on pregnancy outcomes, as well as any gender-related differences in exposure, risk, or vulnerability to climate-related health impacts. This could help situate the review within a wider discussion of gendered health vulnerabilities in the context of climate change.
In the second paragraph of the revised introduction, we have now included a point that links broader impacts of climate change on gender and maternal health and birth outcomes (line 31-33).

Methods / results

- The timing of heat exposure during pregnancy may differentially affect maternal and birth outcomes. For instance, third-trimester exposure has been particularly associated with preterm birth, whereas first-trimester exposure may be more relevant for congenital anomalies. However, it is unclear whether the authors accounted for the timing of heat exposure in their meta-analyses (i.e., whether exposures across different trimesters were combined). Clarifying and contextualising the timing of exposure in the results would enhance the interpretation and help assess potential sources of heterogeneity across studies. If data permit, it would be valuable to disaggregate the analyses by timing of exposure.

Thank you very much for this valuable comment. We fully agree that timing / gestational period is an important factor and would have liked to include it in our meta-analysis. However, the substantial variation in how studies defined and reported gestational timing made such stratification impossible. We have now added a clarifying sentence in the Summary of Effects section (line 124-126), noting that the meta-analysis could not be stratified by gestational period.

For the narrative synthesis, we extracted gestational period information from all included studies and harmonised this data into trimesters, as some studies reported results by gestational weeks while others used trimesters. We hope that including this information in the narrative synthesis provides potential readers with additional insights, particularly since this variable could not be analysed quantitatively.

- It is unclear from the description of the data extraction and meta-analyses whether the effect estimates included from individual studies were consistently those from the most fully adjusted models, crude estimates, or based on another selection criterion. This should be clarified. If possible, it may be worth conducting sensitivity analyses with the crude models.

Thanks for pointing towards the need for clarification here. In the data analysis section (line 508-510) we have added a sentence which explains that we have always included the least-adjusted effect estimates. Furthermore, we have performed a leave-one-study-out sensitivity analyses which has been specified in the summary of effects section, (line 140-142).

- The authors mention that they “excluded effect estimates that were based on various lag days.” Does this mean that only effect estimates based on lag 0 were included? This should be clarified, as it is well established that different lag structures can influence the observed association between heat exposure and health outcomes. Providing a rationale for the choice of lag structure—or specifying how lag-related heterogeneity was handled would be helpful.

Indeed, only lag 0 effect estimates were included, since lag periods varied widely between studies. In the summary of effects section (line 130-131) we have now added another sentence that should clarify this process. Furthermore, in the

limitation section of the discussion (line 364-368), we have also added a sentence, highlighting the exclusion of effect estimates other than lag 0 outcomes, a motivation for why this was done, and the limitation it brings.

- Heat thresholds are known to vary according to individual and environmental factors, such as age, acclimatisation, activity level, humidity, pre-existing health conditions, and access to cooling. These important sources of variation do not appear to be accounted for in the meta-analyses or discussed in the interpretation of results. If individual study effect estimates are disaggregated by population subgroups, it may be valuable to explore sensitivity analyses. Similarly, examining differences across climate zones could offer further insight (the authors do divide study locations into tropical, arid temperatre and continental in line 78-79). For instance, individuals living in Australia are likely to be more physiologically adapted to heat than those in the UK, and therefore may have higher thresholds for adverse health effects. Acknowledging and, if possible, analysing these differences would strengthen the manuscript.
This is a very relevant comment. We would have liked to include these details in the meta-regression but were unable to do so due to an insufficient number of studies eligible for disaggregation (N > 10 for sufficient statistical power). With the additional five sources eligible for the meta-analysis identified through backwards citation chasing, we were able to stratify by climate zone and conduct a meta-regression specific towards the temperate climate zone, where the numbers of studies now are sufficient (N = 10). Unfortunately, the number of studies was not sufficient for other climate zones. Furthermore, we have also disaggregated the analysis into preterm birth (PTB) marking the focus of a specific health outcome, since there was a sufficient number of studies (N = 13). Nonetheless, the remaining individual and environmental parameters lacked consistency across studies to satisfy the requirements for a meta-regression. In the manuscript, we have now further emphasised that this heterogeneity across studies made disaggregated analyses, apart from climate zone and PTB, impossible (line 131-141, line 376-378). We have also added a sentence in the discussion highlighting the need for future research to assess these parameters, and to assess them in a more systematic manner (line 382-385).

- The meta-analyses presented in Figure 3 and described in the Methods appear to combine different maternal and birth outcomes. I have some concerns about the validity of this approach, as different outcomes may have distinct heat thresholds and may be differentially influenced by the timing of exposure during pregnancy. Pooling such heterogeneous outcomes could obscure meaningful differences and reduce the interpretability of the findings. It would be helpful if the authors could justify this approach and, if possible, provide disaggregated analyses by outcome type or by maternal OR neonatal outcomes.
Thank you for raising this very valid comment. As mentioned in the previous version of the manuscript, we did conduct a separate meta regression for preterm birth (PTB) as the number of PTB studies sufficed. Initially, we chose not to include these results in the manuscript, since the PTB-specific thresholds did not differ substantially from the overall results, and due to the reduced number of

studies, we were left with reduced statistical power. However, we have now reintroduced this analysis (and adjusted for the additional studies also mentioned in the above comment), as we agree that it strengthens the interpretability and practical relevance of the findings. At the same time, we have decided to keep the overall outcome as well, since it is the model with most power (due to most studies) and since the PTB specific thresholds only differed minimally from the overall ones.

Regarding your point on maternal vs. neonatal outcomes: Unfortunately, there were too few studies on maternal health alone to justify a separate meta-regression (even after additional studies). For the neonatal health / birth outcomes, given that most birth outcome studies focused specifically on PTB, we found it more meaningful to conduct a PTB-specific analysis rather than a broader birth outcome meta-regression, which regardless would have been dominated by PTB studies. We believe the focus on PTB will further enhance clarity and applicability of the results.

- It would be helpful if the authors included a brief section with key definitions at the beginning of the Methods, clearly specifying how they define "heat threshold" (i.e. what qualifies as a heat exposure for inclusion) and "adverse maternal health and birth outcomes." This would improve clarity and consistency for the reader regarding inclusion criteria. Currently, additional information on the definition of heat and maternal/neonatal health appears later in the "Data analysis" section (line 563 onwards), which may be confusing without earlier clarification.
Good point! We have now moved information from data analysis section into separate section on "Definition of heat, heat threshold, indices and adverse maternal health and birth outcomes" in the beginning of the method section (line 394-436). Also, a specific heat threshold definition was added (line 400-402).

Supplementary appendix Table S1 – Is it correct that, across all included studies, only smoking, BMI, and air pollution were assessed as covariates? Or are these simply the only covariates for which data were extracted? I ask because I would have expected a broader range of confounders to be reported in the original studies, such as maternal age, socioeconomic status, parity, or pre-existing health conditions. It would be great if this can be clarified.
You are correct that a broader range of covariates have been used across studies. Following your comment, we have expanded the scope of covariates for which data was extracted. In the supplementary file 2 sheet 3 in addition to smoking, BMI and air pollution we now include the following additional covariates

- ***Maternal age***
- ***Parity***
- ***Social Economic Status***
- ***Cooling behaviour***
- ***Gestational period***

These are divided into

1. ***Not reported***

2. ***Reported (reported in the study, but not used as a covariate in the analysis)***
3. ***Analysed (used as a covariate / adjusted for in the analysis)***

Gestational age was the only variable for which we specified the outcomes across the included studies. As some studies reported gestational weeks and other studies used trimesters, we synthesised all outcomes into trimesters.

Supplementary Table S2 – The table currently includes an extensive amount of citation information (e.g., full title, DOI, and author list), which is not essential for a study characteristics and results table. Instead, this space could be used more effectively to present key details on individual studies and results. I recommend consolidating the citation information into a single "Study" column (e.g., "Tiwari et al. 2024") and expanding the table to include relevant methodological and outcome-related information such as population source, time period of data collection, exposure definition + acertainment, outcome(s) definition + ascertainment, covariates adjusted for, and main findings. This would enhance the utility of the table, and is particularly relevant for those studies that were not included in the meta-analyses given their results are only very limitedly discussed. The authors can also consider splitting the table up in a study characteristics and a results table.

This is a very valuable advice, which we have chosen to follow. As outlined above, the extensive citation information has been simplified into a single "Study" column. In addition, we have now included the following details (supplementary file 2, sheet 2 and 3):

- ***Title***
- ***Study***
- ***Sample Size***
- ***Aim***
- ***Study Design***
- ***Location***
- ***Climate Zone***
- ***Data Collection Period***
- ***Population Source***
- ***Exposure Definition***
- ***Heat Information***
- ***Outcome***
- ***Main Finding***
- ***Covariates (added in a separate table, due to the tables' extensive size).***

Table 1 – To improve clarity, consider subdividing the "Health outcomes" section into maternal and neonatal outcomes. This would help distinguish which outcomes directly affect the mother versus the child.

Very good point to enhance interpretability of results. The table has now been divided accordingly.

Line 100-102 – The authors mention "additional environmental parameters included," but it is unclear whether this means that temperature variables were jointly assessed with these parameters (e.g., in multi-variable models), or whether an individual effect

estimate for temperature was still reported. Clarifying this distinction would help interpret how temperature-specific effects were isolated and included in the meta-analyses.
A statement was added to clarify that the parameters mentioned are distinct from the specified temperature, since this review focuses on temperature-specific effects (line 95-96). While some studies may have used more comprehensive heat indicators that incorporate these parameters, environmental factors listed separately from heat are not the focus of this review.

Summary of effects section – This section could benefit from a clearer explanation. It would be helpful to understand why such a large number of effect estimates were extracted. Are these based on different model adjustments, lag times, exposure time windows, temperature definitions (e.g., Tmax, Tmin, mean temperature), or other variations within individual studies? Providing this detail would clarify the heterogeneity of the extracted estimates and how they were handled in the analysis.
The large number of effect estimates reflects our approach of extracting as much relevant data as possible, given the uncertainty about which aspects we would ultimately be able to analyse (e.g. several effect estimates for different gestational periods). Consequently, the extracted effect estimates differ between studies, as the study populations, exposure and reported outcomes were not identical. Since we did not restrict our findings, except for only focusing on lag day = 0, we included all effect estimates that met the criteria outlined in lines 111-115. Furthermore, some studies also explored more than one health outcome, or used several heat indicators resulting in several effect estimates for each outcome and heat index. We hope that this explanation now provides sufficient clarity.

Line 502-503 – In addition to referencing the PRISMA checklist, it would be appropriate to also include the MOOSE guidelines for reporting meta-analyses of observational studies: https://jamanetwork.com/journals/jamasurgery/article-abstract/2778476
Thank you for bringing our attention to the MOOSE checklist. The manuscript has been revised accordingly, and the MOOSE checklist has now been completed and added as a separate document to the submission system.

Line 506 – The authors state that all study designs were included; however, if observed correctly, no ecological studies appear to have been included. Were these simply not identified?
We have classified the study designs into the categories defined by the OHAT risk of bias assessment tool, as this was used for the quality assessment of the included studies. The study designs are categorised into:

- ***EA: Experimental Animal***
- ***HCT: Human Controlled Trial***
- ***Co: Cohort***
- ***CaCo: Case-Control***
- ***CrSe: Cross-sectional***
- ***CaS: Case Series/Case report***

Ecological studies are not a category in itself, but below we have listed the studies that would classify as ecological studies:

1. ***Ranjbaran, Ambient temperature and air pollution, and the risk of preterm birth in Tehran, Iran: a time series study***
2. ***Jegasothy, Maternal factors and risk of spontaneous preterm birth due to high ambient temperatures in New South Wales, Australia***
3. ***Song, Short-term effects of ambient temperature on the risk of premature rupture of membranes in Xinxiang, China: A time-series analysis,***
4. ***Mohammadi, Environmental extreme temperature and daily preterm birth in Sabzevar, Iran: A time-series analysis***
5. ***Asta, The modifying role of socioeconomic position and greenness on the short-term effect of heat and air pollution on preterm births in Rome, 2001-2013***
6. ***Arroyo, Impact of air pollution and temperature on adverse birth outcomes: Madrid, 2001-2009***
7. ***Arroyo, Short term effect of air pollution, noise and heat waves on preterm births in Madrid (Spain)***
8. ***Vicedo-Cabrera, Exposure to elevated temperatures and risk of preterm birth in Valencia, Spain***
9. ***Porter, The relation of gestation length to short-term heat stress***
10. ***Khodadadi, Universal Thermal Climate Index (UTCI) and adverse pregnancy outcomes in Ahvaz, Iran.***
11. ***Ranjbaran, Effect of ambient air pollution and temperature on the risk of stillbirth: a distributed lag nonlinear time series analysis***

Line 508-109 – Consider moving the sentence “Studies in a language not spoken by any of the authors were also excluded” to the beginning of the Methods section, alongside a list of the languages spoken by the authors. This would improve clarity while avoiding disruption to the flow of the inclusion/exclusion criteria.
Line 513 - Please specify the languages spoken by the authors to clarify the exclusion criterion: “Studies in a language not spoken by any of the authors were also excluded.”
The sentences have been moved to the beginning of the “search strategy and selection criteria” section, and the languages spoken by the author-team has been added (line 441-444).

Line 519-520 – The selection of databases searched is likely to bias the results toward English-language journals. This potential language and publication bias should be acknowledged as a limitation in the limitations section.
A sentence has now been added acknowledging the English-language and publication bias in the beginning of the limitation section (line 338-340).

Appendix 2 – The search strategy does not include specific terms related to individual maternal or birth outcomes. This raises the concern that relevant studies focusing on particular outcomes (e.g., preeclampsia, gestational diabetes, stillbirth, congenital

anomalies) may have been missed if they did not also include broader terms such as “pregnant” or “postpartum.” Did the authors test whether incorporating specific outcome-related keywords affected the search yield?
Under “search strategy and selection criteria” we have mentioned that within PubMed we have assessed MeSH terms that cover most suitable keywords, and that through this explosion of search terms, we have built the string that was then also used in Web of Science and Scopus. This procedure was done with the help of a librarian. The part in the manuscript has now been somewhat extended to incorporate more detail. We hope that this makes our strategy clearer (line 467-471).

Line 543-544 – It would be helpful to clarify whether the authors conducted double data extraction on a subset of studies to assess inter-rater variability, or whether the data extraction tool was piloted in any way.
The data extraction process has now been described more clearly (line 492-502).

Discussion
- Much of the discussion is centred around the need to develop early warning systems (EWS) for heat-related pregnancy outcomes, but it does not mention any existing tools or initiatives already in development. For example, the HIGH HORIZONS initiative is currently developing the MotherHeat Alert app, which aims to warn pregnant and postpartum individuals—as well as maternity care providers—about rising heat in their area and provide guidance on how to stay safe. Reflecting on such existing or emerging tools (and what thresholds they are based on) would add depth to the discussion and highlight how research findings like those presented in this review could support or refine these real-world applications.
Thank you for this valuable suggestion. We agree that including examples of currently developed system strengthens the discussion. In the revised manuscript we have included a few sentences discussing the MotherHeat Alert and their warning thresholds (line 289-295).

Several of my comments above raise considerations and potential limitations related to the meta-analyses and the interpretation of results—such as the timing of heat exposure during pregnancy, the pooling of different maternal and birth outcomes, and variability in heat thresholds across populations and climates, definitions of temperature. These points may be worth elaborating on more fully in the Discussion section, as they could influence both the robustness and generalisability of the findings.
Specifying these limitations explicitly in the Discussion section strengthens our interpretation and highlights key consideration related to the meta-regression results. We have therefore added a few sentences to the limitation section in the Discussion to address this comment. Thank you for bringing this to our attention (line 376-387).

Reviewer #2 (Remarks to the Author):

I read this manuscript with great interest, as it addresses a timely and important public health topic: defining heat exposure thresholds associated with adverse pregnancy and birth outcomes through a systematic review and meta-analysis. The study has clear relevance to maternal health policy, particularly in the context of climate change. However, several methodological concerns require careful attention before the manuscript can be considered acceptable for publication.

The central statistical approach used to derive odds ratios (OR) for adverse pregnancy outcomes relies on the equation: $Odds\ Ratio\ for\ adverse\ pregnancy\ outcomes$ = e0.39897−0.03937×Tmax+(−0.00094×Tmax2)
Currently, the manuscript provides limited details regarding the selection and validation of this model. Essential information about model development, assumption checking, standard errors, residual diagnostics, and confidence intervals around predicted thresholds is missing. Moreover, this model is built on data from only 14 studies (192 data points) without external or **sensitivity validation**. Greater transparency and comprehensive model diagnostics are necessary to enhance confidence in these critical results.
Dear reviewer, thank you very much for this important comment. We realise that we did not communicate clearly that the presented equation describes the relationship between Tmax and the odds ratio (OR) based on our analysis. We acknowledge that our wording in the manuscript is likely to have led to this confusion. To address this, we have revised the text to clarify that the equation represents our primary outcome rather than that we calculated the OR using it (line 116-117). Figure 2 illustrates the result that would be obtained when solving the equation using the corresponding data points. We hope that these revisions have now made this point clearer.

We have furthermore also conducted a leave-one-study out sensitivity analysis, which has been specified in the summary of effects section, line 141-146).

Additionally, the manuscript defines heat exposure exclusively in terms of daily maximum temperature (Tmax), without considering duration, frequency, or cumulative exposure. Given that prolonged heat exposure and repeated heatwaves are likely to have more significant health implications than single-day exposures, incorporating these dimensions would substantially improve the applicability and real-world relevance of the findings.
We appreciate this important comment. Unfortunately, the substantial inconsistencies across studies, in terms of lag duration, frequency and cumulative exposure definitions, made it impossible for this review to incorporate these parameters into the analyses. We have altered the text slightly, such that it should become clearer to the reader that duration, frequency and cumulative exposure have not been assessed and have highlighted that this review only focuses on lag 0 effects (line 261-262, line 128-131 and line 365-370).

The meta-analysis also reveals substantial statistical heterogeneity (I^2 = 88.2%). Although acknowledged, the authors have not sufficiently explored potential sources of this variability. Conducting subgroup analyses or meta-regressions stratified by climate zone, socioeconomic status, or study design would help clarify the reasons for heterogeneity and increase confidence in combining results into global thresholds.
Thanks for this valuable comment. After revising and disaggregating our analysis into temperate climate zone and preterm birth (PTB) specific meta-analyses (the only two variables with sufficient statistical power to compute subgroup meta-regressions), the statistical heterogeneity has decreased significantly in all three meta-regressions (overall, preterm birth (PTB) and temperate climate zone, please see line 121-122 and 126-128 and figures 2a, 2b, 2c. Generally, the high level of heterogeneity encountered in the review highlights a broader limitation in the existing literature. We hope that future studies adopt a more standardised reporting practice, such that more robust and comprehensible data can be synthesised. This has been highlighted in the discussion section (line 382-385).

The current conversion of relative risks (RR), hazard ratios (HR), and prevalence ratios (PR) into odds ratios (OR) uniformly assumes a baseline risk of 7.5%. The rationale for this assumption is unclear, and no sensitivity analysis has been performed to test its appropriateness. Given the diverse populations and outcomes included, the authors should explicitly address how this assumption may influence their results.
Thank you for the thoughtful and detailed understanding of this topic. We agree that the rationale behind using a 7.5% baseline risk in recalculating effect estimates could be clarified further in the manuscript.

Based on this comment, we have performed a sensitivity analysis of the baseline risk where temperature slope remained positive and highly significant, and thresholds only changed minimally. This has been added to the paragraph in line 537-542.

We have now added some clarifications in the effect size conversion section of the manuscript (line 523-528) explaining why we assumed a 7.5% baseline risk to be suitable for our meta-analysis.

The choice of a 7.5% baseline risk was guided by existing literature on the prevalence of adverse birth outcomes in high income / western countries, which in the context of existing literature includes Europe, Northern America, Central Asia, Australia and New Zealand. As prevalence varies by outcome, the following estimates support our selection:

- ***Preterm birth: <8% in HIC (Ohuma, et al., 2023; doi: 10.1016/S0140-6736(23)00878-4)***
- ***Low birth weight: 7.2% in HIC (Okwaraji, et al., 2024; doi: 10.1016/S0140-6736(23)01198-4)***

Although other maternal health outcomes (e.g. congenital anomalies (Xie et al., 2025: https://doi.org/10.1186/s12889-025-21642-6), preeclampsia (Abalos et al.,

2013: 10.1016/j.ejogrb.2013.05.005)) indicate lower prevalence rates, they are less common in the included literature: only 5 of the 19 studies eligible for meta-analysis assessed different maternal health outcomes. In contrast, 13 of 19 studies focused on preterm birth or low birth weight, both of which have prevalence estimates close to 7.5% in HIC. As most of our included studies have been conducted in geographic contexts covered by HIC, the assumed 7.5% baseline risk most accurately reflects the dominant outcomes and geographic contexts included in this manuscript. Moreover, this approach aligns with other medical meta-analyses that convert effect estimates using a baseline risk of 7.5% (Urquhart, O., et al, 2022; doi: 10.1016/j.adaj.2022.06.003)

Key confounding factors such as maternal BMI, smoking, air pollution, and pre-existing health conditions were noted but not adjusted for in the analyses. Incorporating these important covariates into the analytical framework would significantly strengthen the robustness and interpretability of the findings.
Thank you for this important comment. As noted in our earlier response regarding heterogeneity, this is an important point that we, too, had hoped to address. However, the substantial heterogeneity across studies- also with regards to covariates, both in terms of whether these were assessed, not assessed and if assessed- how so, prevented us from conducting analyses that incorporated these. While we were able to introduce subgroup analyses for the temperate climate zone and preterm birth (PTB) (see comment on heterogeneity) it was unfortunately not feasible to account for the covariates. Nevertheless, we have now expanded the list of covariates extracted (see supplementary file 2 sheet 3). This way we can at least narratively highlight what characteristics and focal points the studies included in this review have.

Furthermore, the manuscript does not discuss or account for cooling behaviours such as air-conditioning use, shaded environments, or workplace ventilation. Given their substantial influence on individual heat exposure, addressing these factors explicitly is essential for translating findings into practical public health recommendations and early warning systems.
Cooling behaviour has now been systematically extracted for all included studies and added to the supplementary file 2, sheet 3. Although some studies included general estimates of cooling behaviours, such as for instance air-condition usage statistics within a specific location, only a single study reported and included cooling behaviour as a covariate in their analysis. This represents an important consideration that should be included in future research.

The studies included are heavily weighted toward high and upper middle-income countries, with only two studies from low-income settings. To enhance global applicability and relevance, greater emphasis should be placed on addressing the geographic imbalance and discussing implications for vulnerable populations in resource-limited settings.

This is a really important point, which we have now further highlighted in the revised manuscript. We added sentences discussing the limited global applicability of the thresholds that have been calculated based on the available literature. We also clarified that the risks estimated in our review are likely underestimated for vulnerable populations in resource-limited regions (line 320-329).

Finally, some relevant studies appear to be omitted, notably Xiong et al. (Nature Communications, 2020) and Mao et al. (Hypertension in Pregnancy, 2023). Including these works would enhance comprehensiveness and reduce potential biases in the synthesis.

Although during the revisions of this manuscript we have included additional studies (N=7) through backwards citation chasing, the abovementioned studies were not included in the manuscript for the following reasons:

1. ***Mao et al. (Hypertension in Pregnancy, 2023) is a review article. Our inclusion criteria is limited to original research studies***
2. ***Xiong et al. (Nature Communications, 2020) does not address the threshold concept and therefore also did not meet the selection criteria for the current study***

We hope the reviewer agrees that these are valid reasons for excluding these papers from the present work.

Reviewer #3 (Remarks to the Author):

Thank you for asking me to review this interesting systematic review and meta-analysis on heat thresholds for maternal health and birth outcomes. The research covers an important area. However, the reporting does not meet current standards in the field which makes assessing the quality of the methods and therefore the robustness of findings challenging. I have therefore limited by comments to the reporting of the systematic review methods as without confidence in these, the analysis/results do not merit further consideration.

1. PRISMA guidelines are guidelines for reporting of systematic reviews and meta-analyses, they are not guidelines for the conduct of a review. Stating that the review has been conducted according to PRISMA guidelines is therefore incorrect. Moreover, although the authors refer to the guidelines they have not followed them in the reporting of the review. The abstract is missing multiple methodological details e.g. data sources, dates of searches, methods of study selection, data extraction and risk of bias assessment etc. etc. Please refer to the PRISMA guidelines for reporting abstracts.
Thank you for this important comment and for noting the incorrect phrasing regarding adherence to PRISMA guidelines. This has now been corrected to state that PRISMA framework was followed for reporting (line 441). To clarify the methodological framework, and following another reviewer's advice, we have now used the MOOSE guidelines were used to conduct the meta-analysis and systematic review, which has been added to the same section (line 439-440). The abstract has been revised according to the PRISMA 2020 abstract guidelines, and the missing details have been added accordingly. In addition, the abstract has been shortened to 150 words to meet the Nature Communications 150-word limit.

2. A systematic review can only ever be as good as the search underlying the selection of included studies. I would expect to see a wider range of databases including some that index environmental journals, and some supplementary search methods e.g. forwards and backwards citation chasing, website searching etc. Also Web of Science is a platform hosting multiple specific databases, it is not correct to say that Web of Science was searched without naming the specific databases that were searched.
Thanks for this very valuable comment which entails many important thoughts. Below we address your three main points in this comment

1. ***Wider range of databases: After consultation with a librarian, we decided not to expand our search to further databases. As specified in the manuscript, we believe that including one medical database (PubMed) and two multidisciplinary databases provides sufficient coverage of journals relevant to our topic.***
2. ***Supplementary search methods: Following further discussion with the librarian, we supplemented our search strategy with backward citation chasing. Through this process we identified seven additional relevant studies, five of which were included in the meta-regression (see fig 3, p. 17).***

3. ***Thank you for highlighting the need to clarify the specific Web of Science configuration used. With the assistance of the librarian, we have now specified that the Web of Science Core Collection was employed. The included indexes are now listed in Table 1 as follows:***

Web of Science Core Collection includes
- ***Science Citation Index Expanded (SCI-EXPANDED),***
- ***Social Sciences Citation Index (SSCI),***
- ***Emerging Sources Citation Index (ESCI),***
- ***Conference Proceedings Citation Index Science (CPCI-S),***
- ***Book Citation Index – Science (BKCI-S),***
- ***Arts & Humanities Citation Index (A&HCI),***
- ***Conference Proceedings Citation Index – Social Science & Humanities (CPCI-SSH),***
- ***Book Citation Index – Social Sciences & Humanities (BKCI-SSH),***
- ***Index Chemicus (IC), Current Chemical Reactions (CCR-EXPANDED)***

This is also specified in the appendix p. 29 (line 848-853)

3. It is disappointing to see the methods section as an appendix, readers can not judge the robustness of the research without sight of the methods and these should be easily accessible.
This comment is addressed together with the comment below. See therefore response for comment 4.

4. The methods section is confused, methods for study selection are described in two different places. It is not clear what is meant by Covidence being used for the synthesis and selection of literature - in what way was Covidence used to synthesise?
Thank you for pointing out the confusion regarding the placement of the Method section. We have now revised the manuscript to follow the standard Nature Communications structure: Abstract, Introduction, Result, Discussion, Method. Accordingly, the Methods section has been moved to follow the Discussion and now includes both the PRISMA 2020 flowchart, and the methodological details previously located in the Appendix.

To clarify the initial structure, we followed the format used by Lakhoo et al., 2024, "A systematic review and meta-analysis of heat exposure impacts on maternal, fetal and neonatal health", which is frequently referenced throughout the manuscript and can be seen to serve as a baseline for our review. However, we agree with your point that presenting the Methods section in a more prominent position makes it more accessible to the readers.

Thank you for your comment regarding the use of Covidence. We agree that our point required more clarification. We have now revised this sentence in the manuscript, specifying that Covidence was used to organise and manage the review process, separating relevant from non-relevant studies across different

screening stages (line 444-445, line 484-486, line 570-571). We hope it has become clearer now.

5. Data extraction was performed by one reviewer and not checked by a second. This is a key limitation. Best practice methods involve two reviewers because errors/differences in interpretation are common in data extraction.
This is a very important point. In the data-analysis section, line 492-502 of the revised manuscript, we have included a more detailed description of our data extraction process that aims to clarify our data extraction. Although data extraction was primarily conducted by a single person, the procedure followed was carefully designed by several co-authors. An extraction framework was piloted using five studies, in which both the primary data extractor and a co-author jointly extracted data to ensure consistency and clarity. Throughout the extraction process the primary extractor maintained ongoing discussions with two co-authors regarding data selection and interpretation. Multiple iterations of data extraction were performed based on feedback from these co-authors, which likely strengthens the accuracy and overall quality of extracted data. In the limitation section of the discussion, we also highlight this limitation (line 355-360).

6. Although risk of bias was assessed - no detail is provided about how many reviewers performed this task, how discrepancies were dealt with or what the impact of the findings was on the synthesis.
Thank you for highlighting this limitation, we have now added a sentence in the data analysis section (line 515-517) to describe this process in more detail. All included articles were assessed by the first author, except for those in a language not spoken by the first author, which were assessed by a co-author fluent in that language. We would also like to point out that the OHAT risk of bias tool is not intended to exclude studies but rather to assess and transparently report their methodological quality. Only two studies were rated as having lower quality compared to the others, however they were retained to ensure a comprehensive synthesis of the available evidence.

SLR & MA NC Revisions II

Reviewer #1 (Remarks to the Author):

Thank you for the revised manuscript. Overall, great work with the revisions! I do have a few more points of clarification and improvement:

- The authors have now clarified that they selected lag0 effect estimates. It should be made much more explicit, ideally in the abstract and early in the methods, that this study focuses specifically on immediate (lag 0) effects on maternal health and birth outcomes. I agree with Reviewer 2 that a major limitation of the meta-analysis is the lack of assessment of duration, frequency, and cumulative exposure, and the restriction to lag 0 effects only. While I appreciate that studies examining duration, frequency, and cumulative exposure may vary in their definitions and reporting, many primary studies typically present results across multiple lag structures or include sensitivity analyses. It would therefore seem feasible to extract these data to conduct additional meta-analyses comparing short-lag and longer-lag effects, or at minimum to provide a clearer justification for restricting the analysis to lag 0 (is it reasonable to assume the effects as this immediate on maternal and neonatal outcomes?). This was also done in the previously published meta-analyses by Lakhoo et al.

Dear reviewer, thank you for bringing up this important point. We agree that assessing lagged effects provides important insights into the relationship between heat exposure and maternal and birth outcomes. We have revised and extended the narrative analysis to include effect estimates across different lag structures, consistently extracting the highest effect estimate reported if several outcomes were presented. This is in line with Chersich et al's 2020 review (often cited throughout the manuscript): "In studies that reported several significant outcomes, we selected the measure with the highest effect size". https://www.healthynewbornnetwork.org/hnn-content/uploads/Climate-change-and-birth-outcomes-BMJ-systematic-review-2020.pdf. Whenever an effect estimate was associated with a specific lag structure, this was explicitly reported alongside the result. For the meta-analysis, however, we decided to restrict our analysis to lag 0 estimates, for methodological consistency and comparability. This is now emphasised in the beginning of the method section, line 461-466 and in the abstract, line 11-12 (and throughout the manuscript).

Lag 0 is the most consistently reported lag across the included studies, whereas other lag structures are highly heterogeneous, including single-day lags (e.g. lag 1, lag 6) and multi-day lag windows (e.g. lag 0-6 or cumulative windows). Selecting lag 0 avoids arbitrary decisions about which lag window to prioritise when multiple estimates are presented within a single study, thereby improving reproducibility and comparability across studies. It also reduces heterogeneity introduced by inconsistent lag definitions. Furthermore, lag-specific results may be selectively reported, with studies highlighting only the largest or statistically significant effects. Focusing on lag 0 further helps minimize potential confounding from time-varying factors (e.g. other environmental exposures, behavioural changes, or socioeconomic and occupational factors), that may introduce noise over longer lag periods. In addition, same-day exposure provides the most temporally precise alignment between exposure and response / outcome.

From a biological perspective, acute responses to heat are well documented and may occur within hours of exposure. Responses such as dehydration, hormonal stress and uterine contractions as well as thermoregulatory strain (e.g. reduced uterine blood flow and vasodilation) support the likelihood of immediate effects rather than delayed responses over several days. Finally, prior systematic reviews from within this field and upon which our work is heavily based and has often cited, have handled lag structures inconsistently. For example, Chersich et al 2020 systematic review which used different lag structures, selected the largest reported effect estimate when multiple lag specifications were available, rather than standardizing lag selection (as briefly outlined above for validation): "In studies that reported several significant outcomes, we selected the measure with the highest effect size. Given that the lag or temperature threshold at which impacts are largest is unknown and will probably vary between populations, the highest estimate might be the true highest level, rather than the result of multiple testing". Lakhoo et al., 2024 systematic review compared broadly defined short- and long-lag windows. These differing approaches highlight the absence of a single best-approach for handling lag windows with this type of literature.

As our work focuses on threshold-based analyses, precise alignment of exposure timing is essential. Effect estimates can vary substantially across individual lag days (see figure below, a recent article which is included in our meta-analysis that presents their effect estimates as mean lag0-6 in the main text, but presents the individual effect estimates per lag day in the supplementary information). Aggregating across broader lag windows risks combining heterogeneous effects into imprecise summary measures. We therefore consider the restriction to lag 0 to be a transparent and methodologically robust approach. We have now adjusted our manuscript to mention more clearly in the abstract an early in the methods (under "definition of heat, heat threshold, heat indices and adverse maternal health and birth outcomes") that the meta-analysis focuses exclusively on acute (lag 0) effects. Also we have added a sentence on the need for future reviews to focus on lagged effects, line 441-443.

Wesselink et al., 2024, supplementary information:

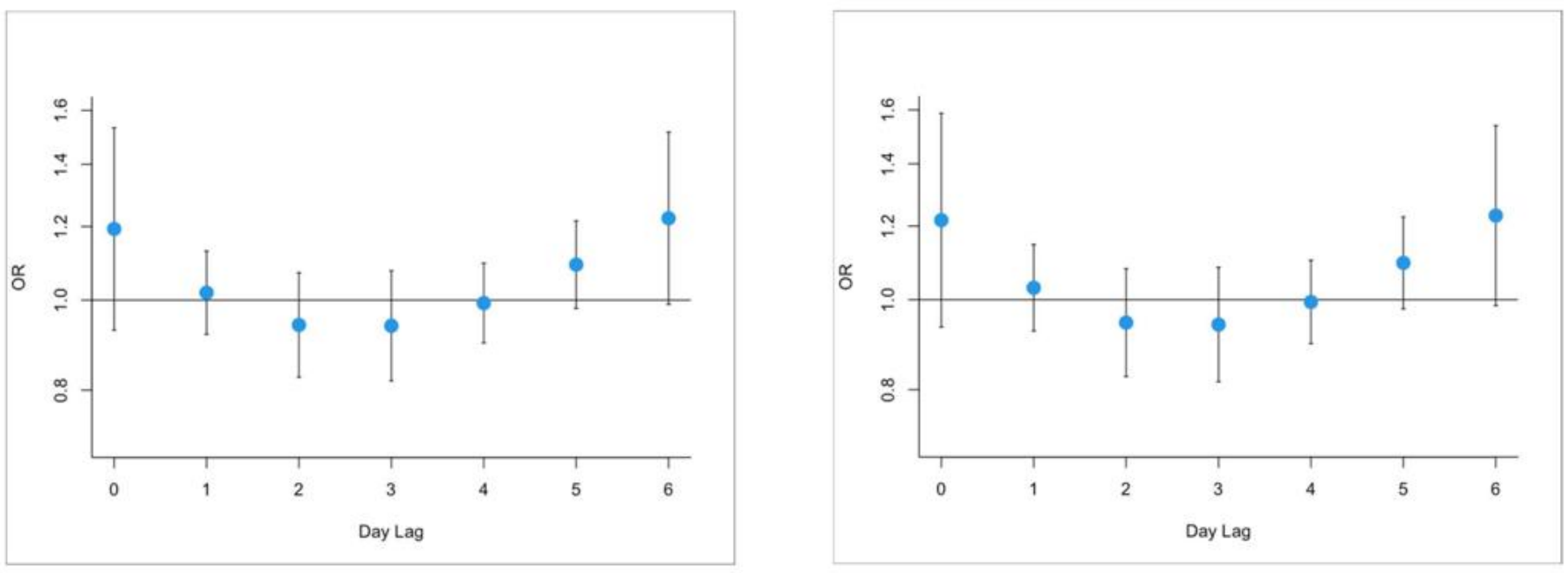

Supplemental Figure 19. Investigation of lagged temperatures over 7 days preceding and including the spontaneous abortion event. The right panel shows the association between temperature and risk of spontaneous abortion when daily maximum temperature percentiles are the exposure of interest; the right panel shows the association between temperature and risk of spontaneous abortion when absolute daily maximum temperatures (Celsius) are the exposure of interest. The graphs display the odds ratios (OR) and 95% confidence intervals (CI) comparing the 95th temperature percentile to the 50th temperature percentile for each lagged day – this corresponds to 19.7 and 32.8 degrees Celsius respectively for the panel on the right. Knots are placed at the 10th, 50th, and 90th temperature percentiles and one internally spaced knot included in the lagged relationship.

- The authors have now clarified that have consistently used the least adjusted models to pool in the meta-analyses "For both the meta-analysis and the narrative analysis, we consistently extracted effect estimates from the ***least-adjusted***

***models**, to minimise discrepancies arising from differences in covariates across adjusted models." This approach is not ideal, as relying on the least-adjusted models is likely to introduce substantial residual confounding. In general, the conventional approach in meta-analyses of observational studies is to extract estimates from the most fully adjusted (i.e., "best") model available, on the assumption that the investigators have selected covariates to minimise confounding and provide the most valid effect estimate. I would therefore encourage you to justify this methodological decision more explicitly, and to change the analyses using the most fully adjusted estimates as primary analyses - potentially alongside sensitivity analyses of the crude models. Have analyses using the "best" models been conducted?*

Dear reviewer, thank you for this important and helpful comment. We agree that in line with standard practice for meta-analyses, extracting effect estimates from the most fully adjusted models is preferred to minimise residual confounding. Our initial approach was to use the least-adjusted models to enhance comparability across studies, since studies vary a lot in covariate adjustments. However, following your suggestion, we have revisited all included studies and revised our approach to consistently extract estimates from the most fully adjusted models available. The methods section has been updated accordingly, and all meta-analyses have been re-run using these estimates. The results and corresponding figures have been updates with no substantial changes to the overall interpretation of the findings. Thank you for your guidance, which we believe has strengthened the methodology and validity of our analyses. Line 584-585 now states we have used the most adjusted model.

*- Although I agree with Reviewer 3 that placement of the **methods within the main text** would have been ideal, the constraints on manuscript length make it appropriate to include the methodological details in the appendix, thereby allowing sufficient space for a thorough discussion of the results. Overall, the clarity of the methodology has improved considerably.*

Dear reviewer, thank you very much for your comment regarding the general structure of the manuscript. In our previous revision round we moved the method section from the appendix to the main text, where it now appears after the discussion section. The methods were originally placed in the appendix, in line with the structure adopted by Lakhoo et al., 2024, however, we agree that including it in the main manuscript improves accessibility and overall clarity. We appreciate your positive feedback that the presentation of the methodology has improved.

- You mentioned that you excluded studies if they were not published in a language that any of the team members were fluent in. I would generally caution against any exclusion of studies based on language given we now have so many translation tools that would support their inclusion. Were there any relevant studies that were excluded based on language? If so, I would reconsider this and include them.

Thank you for this comment, we agree that excluding studies based on language alone is not ideal, particularly given the availability of translation tools. Following your suggestion, we have revisited our screening process to assess whether any of the studies excluded based on language could be eligible for inclusion.

One study met our inclusion criteria for the narrative analysis and has now been incorporated into the review:
"Effects of high temperature and air pollution on the risk of preterm births. Analysis in six Italian cities, 2001-2010". Original title: "Effetti delle alte temperature e dell'inquinamento atmosferico sul rischio di nascita pretermine. Analisi in sei città italiane, 2001-2010", https://epiprev.it/articoli_scientifici/effetti-delle-alte-temperature-e-dell-inquinamento-atmosferico-sul-rischio-di-nascita-pretermine.analisi-in-sei-citta-italiane-2001-2010

- Regarding data extraction, you have partially clarified your approach in your response to Reviewer 3. However, it would be helpful to state more explicitly whether all studies were extracted independently by one reviewer and subsequently checked in full by a second reviewer. While dual independent extraction with consensus is the gold standard, it is acceptable for one reviewer to undertake the extraction (following initial piloting), provided that a second reviewer verifies all extracted data. Given that this is a meta-analysis, the accuracy of the extracted data is critical to the validity of the pooled estimates

Dear reviewer, thank you for this important comment. In line with your suggestion, and that of the other reviewer, we have clarified and strengthened our data extraction procedure. Following revisions, all studies were extracted by one author and subsequently checked in full by other a different author. Prior to these revisions, 20% of studies had been independently checked by a second reviewer. We have updated the Methods section to state explicitly that extracted data was checked and verified in full. We agree that particularly in the context of a meta-analysis, ensuring the accuracy of extracted data is essential for the validity of the estimates. Therefore, we have revised accordingly. We have now clarified our (updated) data extraction process in the data analysis section, line 561 and 573.

*- It looks like something has gone wrong with the **table in the appendix**. I recommend to submit this as a horizontal table to make it readable for the reader. At this point, I can't see which information belongs to which study.*

Thank you for pointing this out, and we apologise for the compromised readability in the appendix table. We have now revised the table layout and reformatted it as a horizontal table to improve clarity and ensure that the information corresponding to each study is clearly presented. We hope the updated version is now easier to read.

Reviewer #3 (Remarks to the Author):

Thank you for asking me to consider the author's response to previous peer review comments. Whilst the author team have made some changes to the manuscript in response to comments, in many places they haven't strengthened the methods underlying the review and as such some concerns remain.

For example, they identified an additional five studies eligible for inclusion in the meta-regression after undertaking backward citation chasing. This represents a quarter of the total included studies in this analysis and could suggest that the ***initial search was not sufficient****. It also indicates that further supplementary searching e.g.* ***forward citation chasing*** *may be necessary.*

Dear reviewer, thank you for your comment. Following your suggestion, we conducted an additional forward citation chasing search to supplement our original strategy.

Forward citation chasing was performed using Google Scholar, as recommended by the specialist in systematic reviews that we consulted, as this database is most exhaustive in its inclusion of sources. This process identified 15 additional sources. Of these, two met the criteria for inclusion in the meta-analysis. Hereof, both studies eligible for meta-analysis were published after the original search date and could have therefore not been identified by the original search (June 2024). Among the 12 studies included in the narrative synthesis, four were published before the original search date and eight were published afterwards. Our original search strategy therefore missed four studies published prior to the search date, whereof none were eligible for the meta-analysis. This supports the robustness of our search strategy for identifying relevant studies. The additional search methods was added both to the method section line 545-550 and to the result section, line 68-69, and we have added the identified additional studies to the analysis and respective results.

Furthermore, the authors suggest that their choice of databases is sufficient - this isn't based on any evidence and with the identification of additional studies through backward citation chasing, may not be correct. I note that one of the other peer reviewers also commented on the limited number of databases used, and questioned the search strategy. Further review of the search strategy by an information specialist with expertise in developing searches for systematic reviews would be advisable.

Dear reviewer, thank you for this important comment. We agree that a comprehensive, well-designed search strategy is critical for systematic reviews. We would like to highlight that the original search strategy and algorithm was developed in collaboration with an experienced librarian, as specified in the manuscript, and that this support has been maintained throughout the revision process, also specified in the last revision round. Following your comment, we re-engaged with the librarian to review and refine the search strategy, to accommodate your suggestions and ultimately improve the quality of our work.

We have now expanded the database coverage to further strengthen the comprehensiveness of the search. We have included searches in GreenFile and Cinahl, with publications until June 2024 (not thereafter) in these additional databases, to match the search performed in the other databases. GreenFile, an environment focused database, rendered no additional studies for inclusion (out of N=194 in total). Through Cinahl, a nursing focused database, we found one additional study for inclusion (out of N=609 in total). This information has now been added to the manuscript, specifically see Figure 3 and Table 1 in the appendix for more detailed information. We believe that these steps have improved the robustness and completeness of our literature search. We hope these additions are to your liking. The low number of additional studies through adding additional databases (both environmental and health related) shows that the databases used for this review are sufficiently robust.

The author's approach to checking data extraction is disappointing and may indicate that the synthesis contains errors.

Dear reviewer, thank you for this remark. Following your comment, we have now revised our data extraction procedure. Data has now been extracted by one author and subsequently checked in full by a different author, rather than checking only a 20% sample. Line 561-573 specifies this.

We would, however, like to highlight that the initial approach, data extraction by one author with partial verification by a second, is often applied in studies from within the field, from within this journal or from within the journal family. See listed studies as examples below. Nonetheless, we have adopted the more rigorous approach in response to your comment to further strengthen the robustness of our methods.

1. https://www.nature.com/articles/s41467-025-59012-w
    - "A single reviewer carried out the data extraction using a pre-established data extraction form, while a second reviewer independently verified the accuracy of 10% of extractions."
2. https://www.nature.com/articles/s41591-024-03395-8
    - "For the updated review, screening of titles and abstracts was done by a single reviewer, but study selection and data extraction were completed by two reviewers." For us it is the other way around
3. https://www.nature.com/articles/s41746-025-02054-7
    - "Two second reviewers (A.M. or M.T.) screened a random 20% of the first reviewer's decisions to assess the reliability."
4. https://www.nature.com/articles/s41380-022-01528-4
    - "one reviewer (AJM) completed extraction while a second (SJL) independently verified a random 20% subset of full-text articles"
5. https://link.springer.com/article/10.1186/s12874-018-0576-9
    - "One reviewer (LMS) extracted all necessary information from the eligible systematic reviews. A second reviewer (JJYN) randomly selected and checked 10% of the extracted systematic reviews for potential errors. Disagreements were resolved by

discussion between the reviewers; in case of no concordance, the authors consulted a third reviewer (HJS)."

6. https://link.springer.com/article/10.1186/s12916-025-04201-z
   - Data were extracted using a bespoke and piloted sheet in MS Excel by a single reviewer with 10% checked by a second reviewer, with additional checking during analysis
7. https://link.springer.com/article/10.1186/s12888-026-07813-8
   - "Data were extracted by one reviewer (FF) and 20% of data were checked by a second reviewer (PW)."
8. https://link.springer.com/article/10.1007/s10903-025-01773-z
   - "Data were extracted by one researcher (SFD) with a random 10% of articles checked by a second reviewer (SM)."
9. https://www.nature.com/articles/s44220-025-00550-z
   - We used the artificial intelligence tool Elicit (elicit.com) to facilitate data extraction121,122, the results of which were exported into Excel and thoroughly checked by C.L.N., and further data extraction was performed manually by C.L.N. where required (for example, from supplementary material). Studies for which data were unable to be extracted via Elicit (*N* = 1)86 were manually extracted by C.L.N. S.V.K. completed data extraction for a random 10% sample and all data extracted were further checked by S.M.K."
10. https://link.springer.com/article/10.1186/s12913-022-08341-3
    - "Study selection: Dual screening of title and abstract was undertaken independently by authors (HM, JK and AP). Author RF resolved any conflicts. Single screening of full text studies was conducted independently by authors, with a random double check of 20% (HM, JK, AP and RF). Data extraction and analysis: Data from each of the included studies were extracted by one of four authors (HM, JK, AP, RF)."
11. https://heathealth.info/wp-content/uploads/s00484-024-02783-6.pdf
    - "One author (CB) extracted these datapoints, and these data were presented to other authors for consensus."

I appreciate the word count restrictions, but the abstract would benefit from inclusion of limitations in the methods used to conduct the review, not only the limitations in the evidence identified. Many of the methodological limitations e.g. limited choice of databases, limited supplementary searching, no systematic checking of data extraction have the potential to undermine confidence in the review findings.

Dear reviewer, thank you for this comment. We agree that including key methodological limitations in the abstract would improve clarity. As we have now addressed limitations related to database selection and supplementary searching, we have not included these in the abstract. However, we have incorporated key review process limitations, by noting that data extraction did not follow a dual independent data extraction approach (but rather a single person extracting, and a second person verifying all extracted data), and that quality assessment was conducted by a single person. We hope the changes are to your liking.
